# Photoinduced electronic and ionic effects in strontium titanate

**DOI:** 10.1039/d1ma00906k

**Published:** 2021-10-26

**Authors:** Matthäus Siebenhofer, Alexander Viernstein, Maximilian Morgenbesser, Jürgen Fleig, Markus Kubicek

**Affiliations:** Institute of Chemical Technologies and Analytics, Vienna University of Technology Austria matthaeus.siebenhofer@tuwien.ac.at markus.kubicek@tuwien.ac.at; CEST Centre of Electrochemistry and Surface Technology, Wr. Neustadt Austria

## Abstract

The interaction of light with solids has been of ever-growing interest for centuries, even more so since the quest for sustainable utilization and storage of solar energy became a major task for industry and research. With SrTiO_3_ being a model material for an extensive exploration of the defect chemistry of mixed conducting perovskite oxides, it has also been a vanguard in advancing the understanding of the interaction between light and the electronic and ionic structure of solids. In the course of these efforts, many phenomena occurring during or subsequent to the illumination of SrTiO_3_ have been investigated. Here, we give an overview of the numerous photoinduced effects in SrTiO_3_ and their inherent connection to electronic structure and defect chemistry. In more detail, advances in the fields of photoconductivity, photoluminescence, photovoltages, photochromism and photocatalysis are summarized and their underlying elemental processes are discussed. In light of recent research, this review also emphasizes the fundamental differences between illuminating SrTiO_3_ either at low temperatures (<RT) or at high temperatures (>200 °C), where in addition to electronic processes, also photoionic interactions become relevant. A survey of the multitude of different processes shows that a profound and comprehensive understanding of the defect chemistry and its alteration under illumination is both vital to optimizing devices and to pushing the boundaries of research and advancing the fundamental understanding of solids.

## Abstract 2

Illumination of SrTiO_3_ induces a multitude of electronic and ionic effects. Recent advances in photoconductivity, photoluminescence, photovoltage, photochromism and photocatalysis are highlighted.

## Graphical abstract

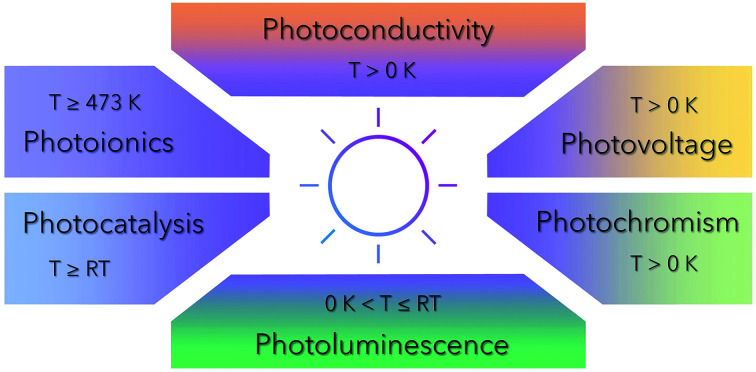

## 1 Introduction

Strontium titanate (SrTiO_3_, STO) is a perovskite-type oxide and has long been in the focus of basic and applied research due to its broad variety of well-investigated properties and multiple fields of application.^1–3^ It was the first semiconducting and (when lightly doped) the first oxide perovskite superconductor, it shows an unusually high dielectric permittivity at low temperatures and undergoes a characteristic tetragonal to cubic phase transition at around 105 K.^4–6^ STO single crystals are commonly available and often used as a substrate for the epitaxial growth of other perovskite thin films. STO thin films themselves also exhibit many interesting properties *e.g.* for memristive devices.^7,8^ Recent research has also focussed on its surface properties, ranging from the formation of remarkably conductive interfaces^9,10^ to highly interesting photoresponses.^11–14^ Especially with regard to its photoactive properties, STO is an essential base material to advance the insight into the fundamental physics for a wide range of applications. This applies particularly to the quest for clean energy conversion techniques, where STO covers several fields of interest, from photoelectrolysis^15,16^ and photovoltaic cells^17,18^ to solid oxide fuel cells (SOFCs).^19,20^ This versatility and the comparatively good understanding of the material makes it an ideal model material for researchers of various disciplines and fields of interest.

This review focuses on the aforementioned photoactive properties of STO and gives an overview of the plethora of different effects arising upon irradiation. By this work, we want to categorize the various reported effects and analyze the mechanisms behind different immediate or persistent responses to illumination. A detailed understanding of the underlying processes causing these effects is crucial not only for the optimization of materials and photoactive devices but especially with regard to the utilization of solar energy and the design of novel applications based on this clean energy source. The need for a broad survey of photoinduced effects on STO emerges from their great diversity and also from the fact that depending on the experimental conditions, interactions of STO with above band gap radiation may be fundamentally different while inducing seemingly similar effects. At low temperatures, excitation and recombination processes of photogenerated charge carriers and thus the electronic energy landscape of STO are in the center of attention. Studies at elevated temperatures focus on surface reactions and on the alteration of oxygen exchange kinetics by UV illumination.^13,14,21–25^ Although the interaction is different, both of these phenomena can for example be manifested in enhanced conductivity of STO persisting even after the illumination.^14,25,26^ This article tries to bridge this gap and gives a conclusive summary of the various effects triggered by the interaction of light with STO at different temperatures. It further aims to illustrate their differences and similarities in a comprehensible manner across the various research communities involved to promote an interdisciplinary understanding of the underlying physics and to advance on the path to a broad and effective application of photoinduced effects in devices across the fields of sensors, catalysis, and fuel cells.

## 2 Strontium titanate – a model perovskite

### 2.1 Structure and defect chemistry

**Fig. 1:**
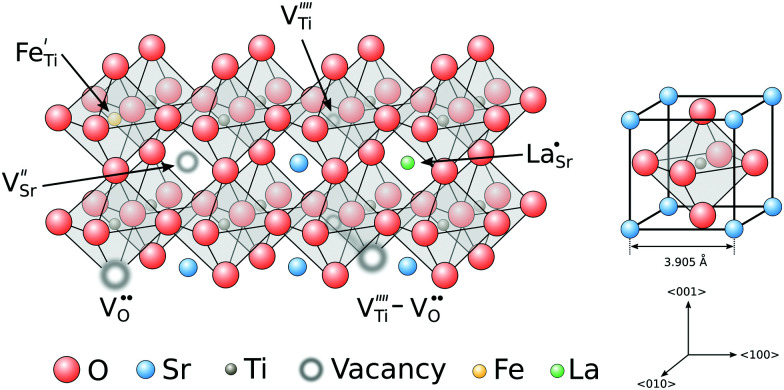
Schematic structure of strontium titanate with different defects integrated in the lattice. All defect species are written according to the Kröger–Vink notation.^49^

Strontium titanate is a typical perovskite material with an ABO_3_ structure built from TiO_6_ octahedra cornered by 8 strontium atoms (Fig. 1). While the structure of STO is cubic above a critical temperature of ≈105 K, a phase transition to a tetragonal structure occurs when the material is cooled below that temperature, leading to tilted oxygen octahedra rotated around the titanium ions.^27,28^ At room temperature, pure STO is transparent and exhibits a lattice constant of 3.905 Å.^29^

From a defect chemical perspective, a variety of defects can occur in pure strontium titanate. While there is sufficient evidence for the existence of long-range disorder like edge and screw dislocations as well as planar defects,^30–33^ they are stationary defects under many experimental conditions. Point defects on the other are largely responsible for mass and charge transport in STO. Both their mobilities and also their concentrations depend strongly on the sample environment and can vary over a wide range of equilibrium conditions.^34,35^

Such point defects in perovskites may appear either in the form of vacancies or as interstitials, however vacancies in general are energetically favoured.^36–38^ Based on the defect site, vacancies can be divided into cation and anion vacancies. Cation vacancies are generally considered immobile until temperatures above 1000 °C and are usually formed to a certain extent during crystal growth or sintering/annealing due to Schottky equilibria.^39,40^ Below this temperature, cation vacancies are frozen stationary and act as acceptor dopants. For oxygen vacancies, which also are inherently present in STO to compensate the aforementioned cation vacancies, the critical temperature above which the defect concentration is frozen, is much lower at around 300 °C.^41^ At higher temperatures, the concentration of oxygen vacancies is in equilibrium with the surrounding oxygen-containing (O_2_, H_2_O, CO, *etc.*) atmosphere, provided that the surface exchange reaction is sufficiently fast. Additional oxygen vacancies, introduced at low oxygen partial pressures, are compensated by the generation of two conduction band electrons respectively, while filling of a vacancy at high oxygen partial pressures generates two electron holes. The electronic conductivity of pure strontium titanate can thus vary over the whole range from n-type conduction in reducing conditions *via* an intrinsic conductivity minimum to p-type conduction in oxidizing conditions.^34,42^ The minimum is often superposed with an ionic (oxygen vacancy) conductivity plateau. Furthermore, impurity defects can be introduced during crystal growth or ceramic preparation. Depending on their valence state they may either be charge neutral or may act as donor or acceptor, respectively. Commonly found impurities in nominally pure SrTiO_3_ include Al, Ba, Ca, Cr, Fe, Mg^43,44^ (mostly in low ppm or sub-ppm range). While these defects are mostly independently present in the lattice at higher temperatures, defect associates between cation impurities and oxygen vacancies as well as clusters containing different defects have been reported at lower temperatures.^45–48^ Importantly, defects may create electronic energy levels in the STO band gap and can thus be part of different mechanisms when exposed to irradiation.

### 2.2 Band gap and doping effects

**Fig. 2:**
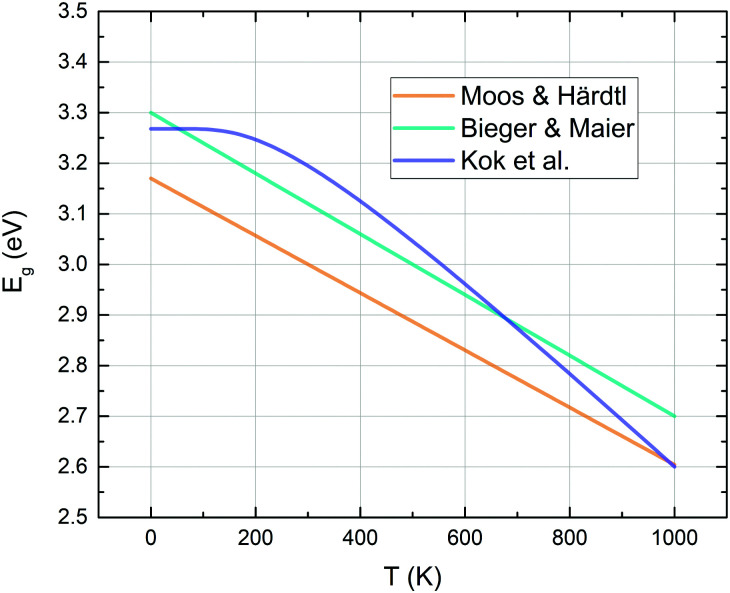
Indirect band gap energies of strontium titanate – curves calculated with the fit formulae proposed by different authors.^34,54,55^

Strontium titanate is known as a wide band gap semiconductor with an indirect band gap of 3.20–3.25 eV and a direct band gap of around 3.75 eV.^50,51^ The top of the valence band mainly consists of O_2p_ orbitals, while the bottom of the conduction band is primarily formed by Ti_3d_ t_2g_ orbitals.^52,53^ The band gap of STO decreases significantly with temperature, as shown in Fig. 2^34,54,55^ leading to increased numbers of mobile electronic charge carriers at higher temperatures.

As the large band gap limits the versatility of STO with regard to absorption characteristics, STO has been doped with various elements to tailor the material for different purposes, for example for photocatalysis. Slight B-site doping of SrTi_1−*x*_M_*x*_O (*x* = 0.05) with Fe, Mn or Co has been calculated to lower the band gap down to 2.1 eV,^56^ other experiments showed a reduction down to 2.3 eV in STO thin films doped with La, Cr and Co.^57^ Contrary effects can be achieved by replacing Ti atoms with Al and consequently increasing the band gap to 3.5 eV (compared to undoped STO).^58^ Apart from photocatalysis, STO is commonly doped to tailor its electronic properties, often for applications at high temperatures. Acceptor dopants like 
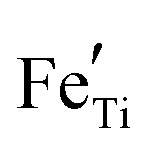
 are used to control conductivity and dielectric behaviour of STO and to overpower the effects of inherently present unwanted background impurities.^59,60^ On the other hand, donor doping with La or Y on the A-site or Nb on the B-site can turn STO into an n-type (semi)conducting material.^61^ Further effects correlated with doping of STO are photochromism when doped with Mo and other transition metals or at high temperatures when doped with Fe^22,62^ or bistable resistance states when doped with Cr.^63^

### 2.3 Basic interactions with light

**Fig. 3:**
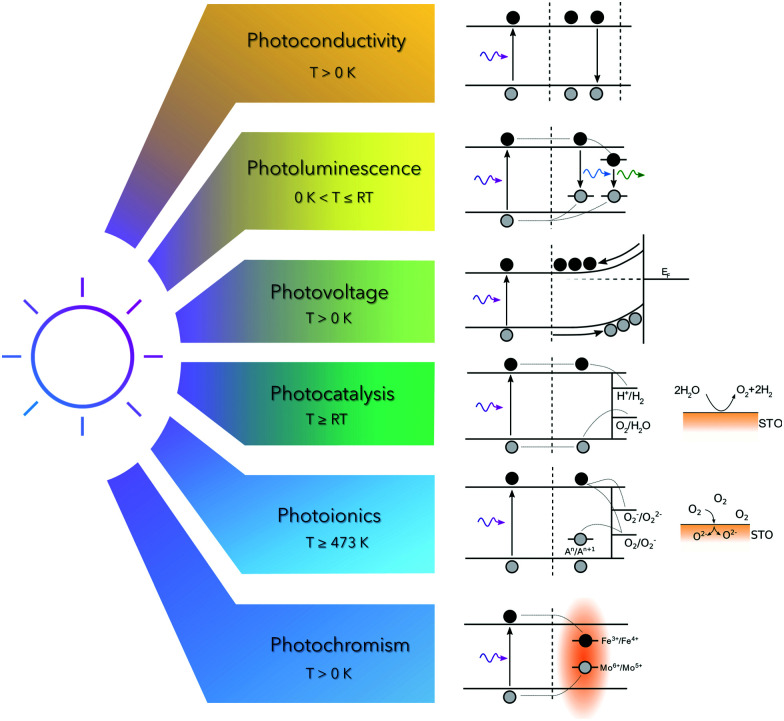
Basic mechanisms subsequent to irradiation with ultraviolet light in strontium titanate at low and high temperatures.

The fundamental process occurring in STO subsequent to irradiation with above-band gap light is the excitation of electrons from the valence band to the conduction band. This causes a variety of different effects, *e.g.* photoconductivity, photoluminescence, photovoltage, photocatalysis and photochromism and can further even induce bulk stoichiometry changes. All these phenomena are discussed in this review and are summarized in Fig. 3.

## 3 Photoconductivity

The discovery of photoconductivity dates back to 1873, when W. Smith investigated the conductivity of selenium and observed that its resistance was altered depending on the intensity of incident light.^64^ Since then photoconductivity has been an ever-growing and active field of research.^65^

### Low temperature effects

**Fig. 4:**
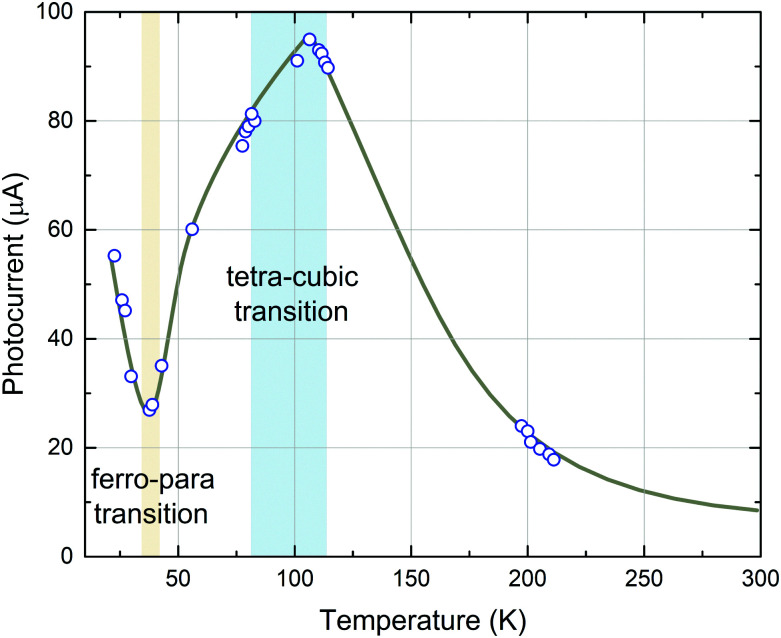
Photocurrent in STO as a function of temperature. Applied potential was 22.5 V and the measured dark currents were 1.2 × 10^−12^ A at 300 K and less than 10^−14^ A at 77 K. Reprinted from ref. 69, with the permission of AIP Publishing.

In STO, photoconductivity was investigated in the 1960s by Yasunaga *et al.*,^66^ who observed a distinct increase of photocurrent when STO is illuminated with light exhibiting a wavelength shorter than 385 nm, (>3.22 eV, around the band gap energy of STO) and concluded that this is caused by n-type electronic conduction due to photogenerated charge carriers.^66^ The extent of this effect increased with decreasing temperature (measured down to 80 K in ref. 66). During extended observations, an anomaly in the temperature dependence of the photoconductivity at 47 K was discovered and the authors assumed local ferroelectric transitions to be the cause of this effect.^67,68^ Sihvonen observed a similar anomaly in the form of a photocurrent minimum at 35 K^69^ and also correlated this effect with a ferroelectric–paraelectric transition (Fig. 4). Furthermore, a second maximum anomaly of the photocurrent at around 100 K was found, which coincides with the tetragonal–cubic transition temperature of STO.^69^ Upon further increasing the temperature, the photocurrent exhibits a continuous decrease.

**Fig. 5:**
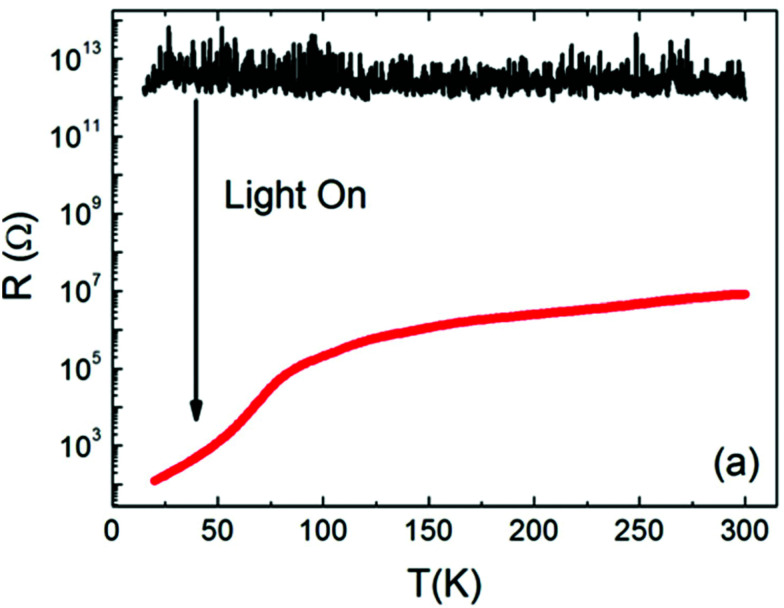
The temperature dependence of the resistance of pure/undoped STO single crystals with and without irradiation. Reprinted from ref. 72, with the permission of AIP Publishing.

A similar trend for the photoconductivity above this transition temperature at 100 K was also found by Katsu *et al.*, however, no anomaly was observed and the conductivity continued to increase sharply when further decreasing the temperature.^70^ The same behaviour was also observed by Zhang *et al.* who found a sudden resistance drop when cooling below 105 K (and again detected an anomaly at around 35 K).^71^ They conclude that this drop is caused by a transition to a direct 3.2 eV band gap in the tetragonal structure.^71^ While Jin *et al.* found very similar results and moreover quantified the conductivity increase with 6 orders of magnitude at room temperature (see Fig. 5),^72^ other recent studies were not able to reproduce this anomalous behaviour in nominally pure STO single crystals. Instead, they found a continuous increase or small local maxima of the photoconductivity when cooling from room temperature.^73,74^ Due to the variety of samples investigated in the previously mentioned studies, it is likely that experimental conditions and especially defect chemical diversity among the investigated single crystals play a substantial role in the observed differences. Possibly, as the defect structure of different single crystals is very sensitive to the exact growth conditions, this may affect the structural transitions and thus also the photoconductivity response of the material.

**Fig. 6:**
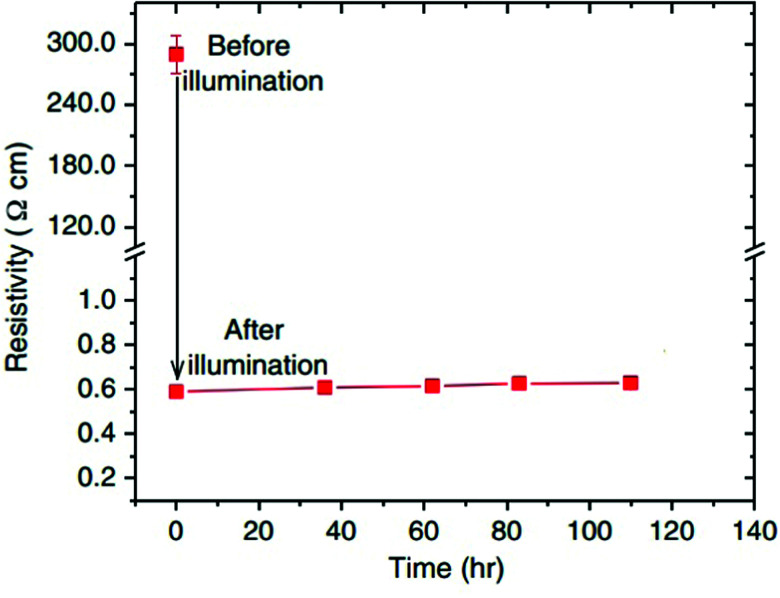
Resistivity of an annealed sample before and after illumination at room temperature. After illumination, the sample was kept in the dark. Reprinted with permission from ref. 26. Copyright 2013 by the American Physical Society.

Jin *et al.* also observed, that depending on the temperature, the decay time of the enhanced conductivity back to its original state varies significantly.^72^ In general, the persistence of photoconductivity has been studied intensively during the last decade. Tarun *et al.* first reported an annealing routine which altered STO samples in a way that their conductivity, enhanced during illumination, remains high at room temperature even days after illumination (Fig. 6).^26^ The importance of this annealing routine was later illustrated by Saadatkia *et al.* who could not observe persistent effects in any of several as-purchased single crystals.^75^ Supported by positron annihilation lifetime spectroscopy (PALS), a new defect type was found after the annealing step, most probably a Ti–O vacancy pair.^26^ The authors argue, that the persistent photoconductivity (PPC) of their samples is caused by photoexcitation of electrons from newly generated defect levels and emphasize the general importance of ionic defects for photoconductivity in STO.^26,76^ Poole *et al.* found that oxygen vacancies and possibly hydrogen impurities are essential for whether photoconductivity persists after illumination or not.^77^ Further experiments indicated that illumination may force hydrogen to leave substitutional sites in the lattice and form H–O bonds, thus liberating additional electrons, contributing to PPC.^78^ DFT calculations of Zhang *et al.* support and further refine this theory of the importance of hydrogen for persistent photoconductivity.^79^ The phenomenon was recently also brought to applicability-level by writing low-resistance paths on STO single crystals employing selective illumination with a laser.^80^

Photoconductivity was also observed in STO thin films and in heterostructures based on STO. Park *et al.* examined epitaxial STO thin films grown with pulsed laser deposition (PLD) and found a current enhancement of around three orders of magnitude under illumination at room temperature.^81^ Xing *et al.* found a similar enhancement factor in polycrystalline STO thin films grown by magnetron sputtering.^82^ With regard to the photoconductivity of STO-based heterostructures, possibly the most striking example is a conductivity increase of around 5 orders of magnitude observed during the illumination of the LAO/STO interface at room temperature.^83^ A similar effect was found by Li *et al.*,^84^ whose experiments also indicated that the electro-migration of oxygen vacancies is significantly accelerated under UV illumination. For further details on the photoresponse of STO-based heterostructures the reader is referred to other review articles.^85,86^

### High temperature effects

**Fig. 7:**
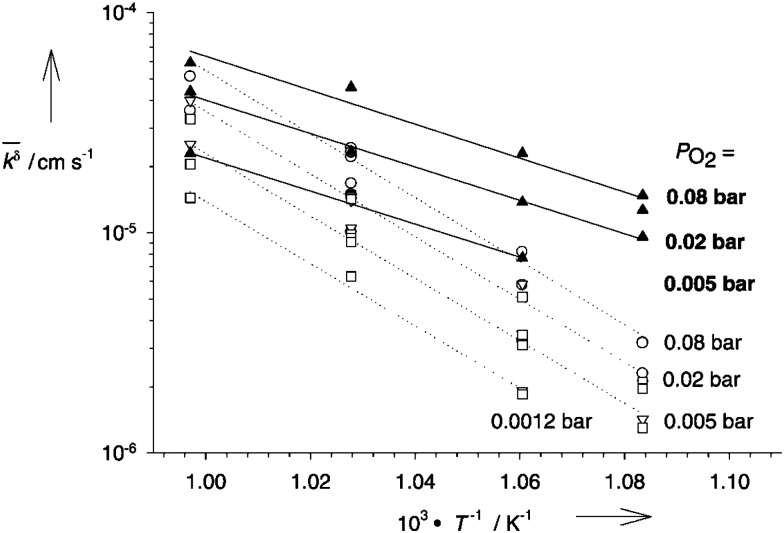
Arrhenius plots of the effective rate constants for oxygen incorporation (solid circles) and release (solid squares) without UV illumination as well as oxygen in- (solid triangles) and excorporation (open triangles) under UV light. Reprinted from ref. 13, with the permission of John Wiley and Sons.

Enhanced conductivity in STO single crystals during and after illumination was also observed at elevated temperatures, but based on a completely different mechanism. The underlying effect was first described by Merkle *et al.* who discovered that the oxygen incorporation rate of STO is increased significantly under UV illumination (see Fig. 7).^13^ As a result of this process, oxygen in STO is subject to a different chemical potential and a new defect chemical state is established. For every additional oxygen ion incorporated into the lattice, two electron holes are generated due to charge neutrality, which enhances the electronic conductivity of p-type STO.

**Fig. 8:**
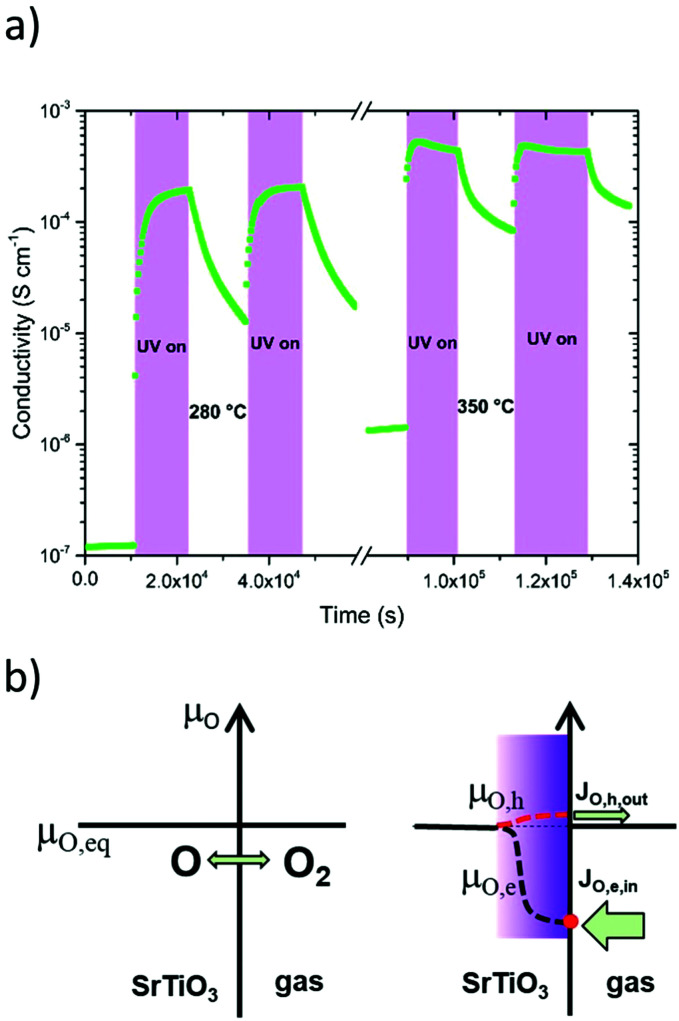
(a) In-plane conductivity changes of an undoped STO single crystal under UV illumination at 280 and 350 °C. (b) Introduction of electron- and hole-related oxygen quasi-chemical potentials under UV irradiation. The red broken line indicates the diffusion dominating quasi-chemical potential (hole related) and the red circle indicates the dominating surface process (electron related).

The direct impact of this process on the bulk resistance of STO was first shown by Walch *et al.* who illuminated an undoped STO single crystal with UV light and observed an across-plane resistance drop of nearly two orders of magnitude.^14,24^ This drop recovers slowly over minutes or hours after turning the UV light off. In contrast to low-*T* effects, however, this persistent increase of conductivity is due to the kinetic limitations of the oxygen surface exchange reaction preventing immediate equilibration. More recent in-plane conductivity measurements on STO single crystals by Viernstein *et al.*^87^ confirmed these effects and refined the underlying model with the introduction of oxygen quasi-chemical potentials induced by UV irradiation, which are responsible for altered diffusion and surface exchange dynamics in STO (see Fig. 8). Similar experiments were also performed on Fe doped STO and an increase of the bulk conductivity under UV light was observed, however, the process was slower compared to nominally undoped STO and the conductivity did not reach a maximum value even after illuminating for several hours.^22^ Likewise, the conductivity increase persisted for much longer than in undoped STO. Time dependences of such stoichiometry-driven conductivity changes could be related to the oxygen chemical diffusion coefficient. This shows that, also at high temperatures, defect types and concentrations play a major role in how UV light affects the oxygen surface exchange and thus the equilibrium defect chemistry and conductivity of STO.

**Fig. 9:**
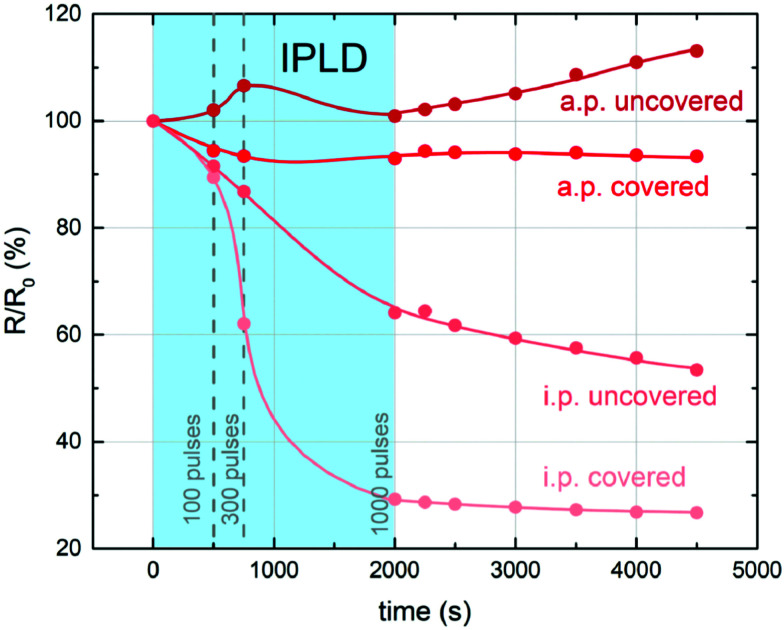
Evolution of the bulk resistance for representative in-plane (i.p.) and across-plane (a.p.) measurements of covered and uncovered STO single crystals during and after illumination/deposition at pulsed laser deposition. Reprinted from ref. 25, Copyright 2021, with permission from Elsevier.

Recent studies also show, that stoichiometry changes do not only occur under deliberate UV illumination, but also during pulsed laser deposition (PLD) processes, where the plasma plume emits radiation in the UV range. Hensling *et al.* reported that the UV irradiation by the PLD plasma plume at 800 °C and 10^−5^ mbar *p*(O_2_) causes an enhanced oxygen vacancy incorporation rate which results in a fully altered defect chemistry of the sample which persists for over 50 days, when quenching an STO single crystal after the PLD process.^21^ In a further study, impedance spectroscopy during pulsed laser deposition (i-PLD) at 300 °C and 0.06 mbar lead to an increased oxygen uptake under irradiation by the plasma plume and hence, again in an increased conductivity of the STO single crystalline substrate, which persists after irradiation (see Fig. 9).^25^ When actual material was deposited on the single crystal in the same study, the resistance change indicated that the growing film induces competing mechanisms which alter the substrate stoichiometry even further and lead to a tri-layer system regarding the oxygen stoichiometry in the single crystal.

The variety of the effects observed during UV illumination of STO at elevated temperatures illustrates that the impact of UV radiation is quantitatively and qualitatively different depending on the current defect chemistry and equilibrium state of STO. Therefore, this phenomenon needs systematic investigation over a wide temperature and oxygen partial pressure range in the future. However, it is clear, that UV illumination and the accompanying near-surface generation of charge carriers have a severe impact on the oxygen surface exchange reaction and can alter the stoichiometry and thus the electric properties of STO significantly.

## 4 Photoluminescence

Photoluminescence is the emission of electromagnetic radiation from a material subsequent to the absorption of photons. It is based on the excitation of charge carriers by photons and recombination processes leading to re-emission of photons of characteristic wavelengths.^88^

### Low temperature effects

**Fig. 10:**
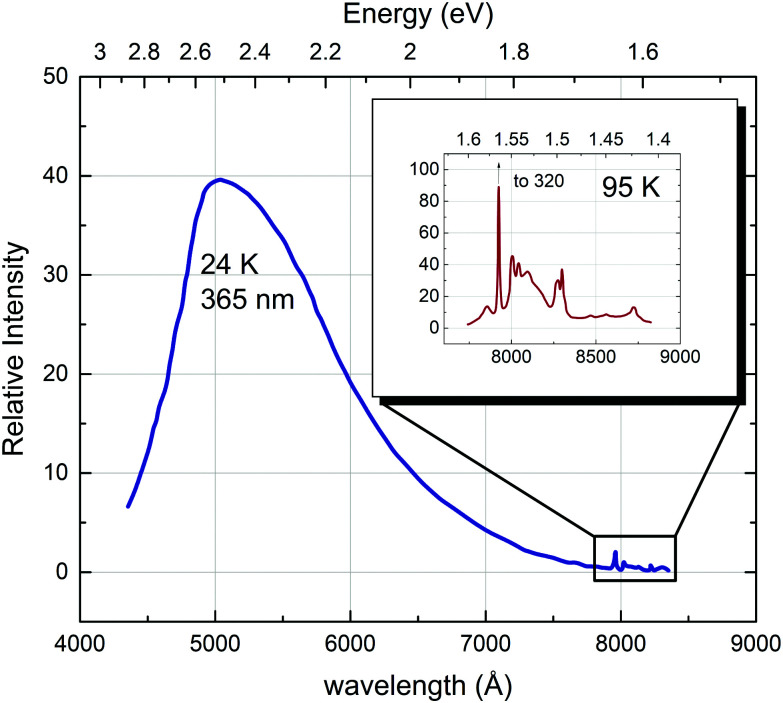
Photoluminescence spectra with recognisable bands in the blue and in the infrared region (detailed view in the inset) recorded at 24 and 95 K, respectively, with an incidence photon wavelength of 365 nm (3.40 eV). Reprinted from ref. 69, with the permission of AIP Publishing.

In strontium titanate, among the first observed photoluminescence effects were a rather sharp infrared emission band and a broad band blue/green photoluminescence:^69^ Sihvonen observed a structured emission band in the infrared region between 20 and 200 K with several clearly distinguishable sharp peaks. The lower the temperature, the clearer appears an additional very broad emission band in the visible and near infrared region (see Fig. 10). The sharp emissions in the infrared region have since been convincingly related to the ^2^E_g_ → ^4^A_2g_ transition of Cr^3+^ ions present either through doping or as undesired impurities, where the sharp peak right below 800 nm corresponds to the zero-phonon line. The other lower peaks are vibronic transitions associated with lattice phonons.^44,89–91^ Feng proposed the following mechanism for the Cr^3+^ transition:^89^

1 Cr^3+^ + e′ + h˙ → Cr^4+^ + e′ → (Cr^3+^)* → Cr^3+^ + *hν* (7930 Å + vibronics),

where (Cr^3+^)* denotes the excited state of the Cr ion. This emission has a maximum intensity at around 100–120 K. At higher temperatures, radiationless decay processes become more important, thus decreasing the intensity of the photoluminescence. At lower temperatures, the intensity is reduced because the electron hole needed for the charge transfer is more likely to get trapped at a sensitizing center in the vicinity of the Cr ion.^89^

**Fig. 11:**
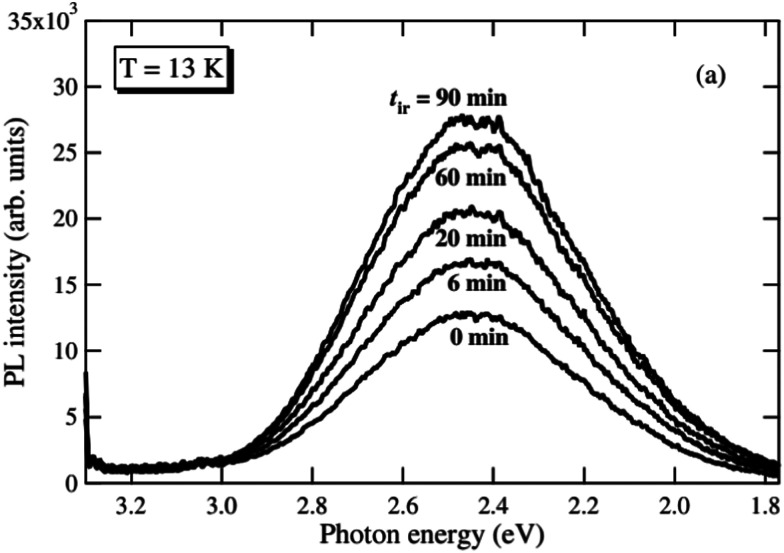
Photoluminescence spectra recorded at 13 K after different annealing times in vacuum under 325 nm laser light irradiation.^97^ Reprinted by permission from IOP Publishing (2005).

The broad emission band in the visible region is commonly related to the recombination of trapped charge carriers.^92,93^ Leonelli and Brebner found a broad emission band with a maximum at 2.44 eV and proposed that its origin is intrinsic due to the recombination of so called self trapped excitons (STEs). Such an STE is an ensemble of a small polaronic electron trapped in a strong lattice distortion interacting with an electron hole.^92,94^ Luminescence decay measurements suggest two decay processes with different timescales. Accordingly, the STE may either be formed directly upon irradiation or retarded from a small polaronic electron interacting with a hole already trapped near other defects or impurities.^92^ Another plausible mechanism is presented by Vikhnin *et al.* who point out that the emission band can also be associated to vibronic charge transfer excitons (VCTEs), a pair of Jahn–Teller electron and electron hole polarons localized on two neighbouring atoms.^95^ This bipolaron could also be trapped at oxygen vacancies.^95,96^ This is also a possible explanation for the results of Mochizuki *et al.* who observed that the intensity of a broad green emission band increases significantly when the sample is annealed in vacuum (see Fig. 11) and reversibly decreases when measured in pure oxygen atmosphere.^97,98^ The authors suggest that UV irradiation strongly affects the surface chemistry of the single crystal and introduces oxygen vacancies when applied in vacuum, even at room temperature. These vacancies (either alone or as defect complexes) tend to trap electrons generated during illumination and could act as trapping centers for the aforementioned excitons.^97^ However, the exact nature of the trapped excitons responsible for this green emission band is still not completely clarified.

**Fig. 12:**
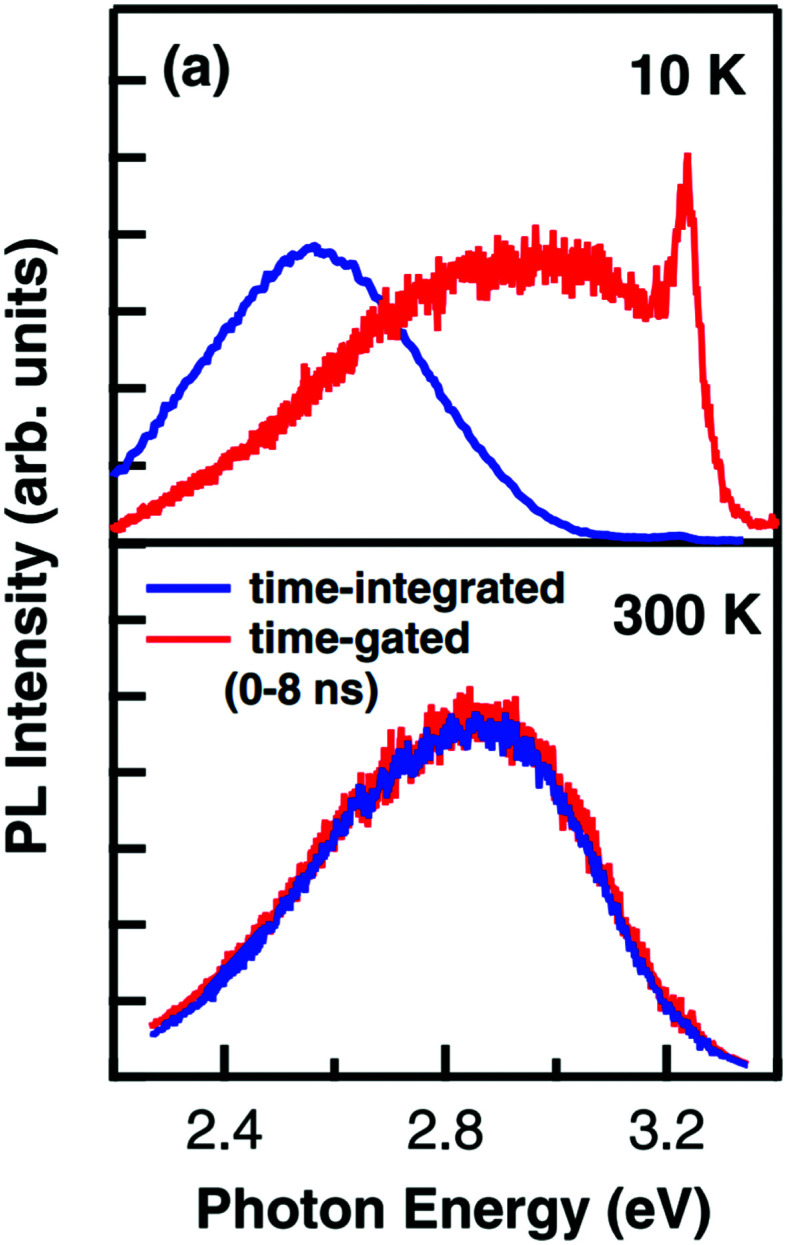
Time-gated and time-integrated photoluminescence spectra measured with high excitation intensity at 10 and 300 K. While the time-integrated spectrum clearly shows the green emission band at low temperatures, the time-gated measurement reveals further emission bands at 2.9 and 3.2 eV. Reprinted with permission from ref. 99. Copyright 2009 by the American Physical Society.

To separate intrinsic phenomena from processes related to extrinsic impurities, high intensity irradiation experiments have been performed to achieve much higher concentrations of photogenerated charge carriers, thus exceeding effects from extrinsic impurities.^97,99–101^ Another broad band emission at higher photon energies (around 2.8 to 2.9 eV) and a sharp peak at 3.2 eV near the band gap energy with much faster decay times were found (see Fig. 12). The blue emission band also persisted at higher temperatures. While the 3.2 eV peak is generally ascribed to radiative indirect band-to-band recombination processes,^102^ the broad 2.9 eV emission has since been discussed as the result of a combination of single carrier trapping processes, bimolecular recombination processes and Auger recombination processes.^100,101^ However, also here, the exact nature of these processes is yet unclear on the atomic scale, particularly with regard to potentially important defects, affecting the recombination dynamics.^101^

**Fig. 13:**
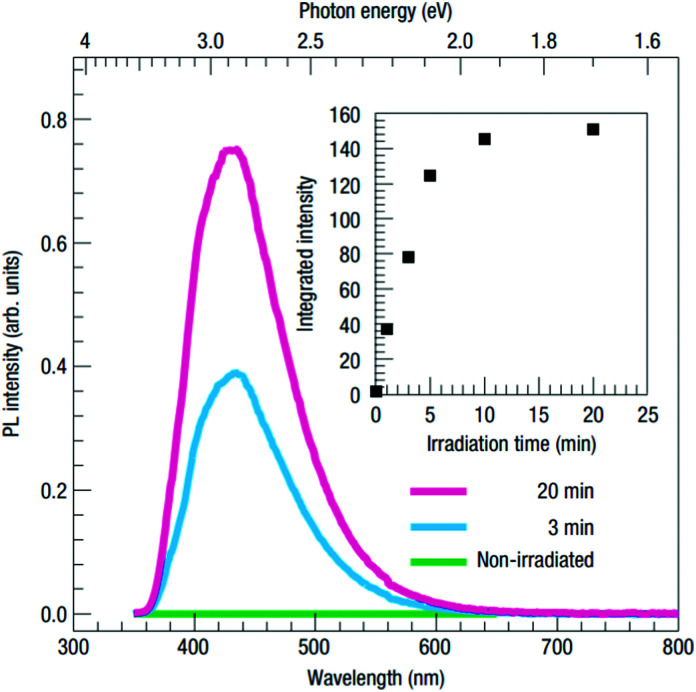
Photoluminescence spectra of Ar^+^ irradiated STO measured for different irradiation times at room temperature. The inset shows the intensity increasing with irradiation time.^103^ Reprinted by permission from Nature materials (2005).

A similar blue-light emission has also been observed in samples with significantly increased electron concentrations *via* electron donors such as La and Nb. Additionally, a large number of oxygen vacancies in near-surface regions are created by the irradiation of STO with Ar^+^ ions, resulting in locally heavily increased electron densities.^103–105^ Kan *et al.* found that Ar^+^ irradiated single crystals exhibit a very thin amorphous top layer and a significant oxygen deficiency about 20 nm underneath.^103,106^ The blue luminescence observed in such crystals at room temperature increases with irradiation time (see Fig. 13). They proposed that the oxygen vacancies facilitate the formation of STEs in their vicinity and that the recombination of holes in such STE states with abundant conduction band electrons causes the 2.8 eV emission.^103,107^ The importance of the oxygen vacancies is, however, subject to an ongoing discussion, as studies on ion beam induced luminescence in STO suggest that isolated oxygen vacancies are not an essential factor in the origin of the 2.8 eV emission band, but rather cause a different, red emission in connection with Ti^3+^ polarons in their vicinity.^108,109^

Essentially the same emission band was also found in donor doped STO which, similarly to Ar^+^ irradiated STO, contains additional conduction band electrons, in this case introduced by La^3+^ and Nb^5+^ at the Sr^2+^ and Ti^4+^ site respectively.^100,104^ While these results also support the conclusion that the blue emission originates from the recombination of a hole in a mid-gap state with a conduction band electron, there is however still a dispute on the nature of the charge carrier trap levels responsible for this emission and whether those are related with point defects or with more complex or even structural defects.^110,111^ In conclusion, it seems established that high conduction band electron concentrations in combination with yet unspecified defects are necessary to observe this blue photoluminescence band.

**Fig. 14:**
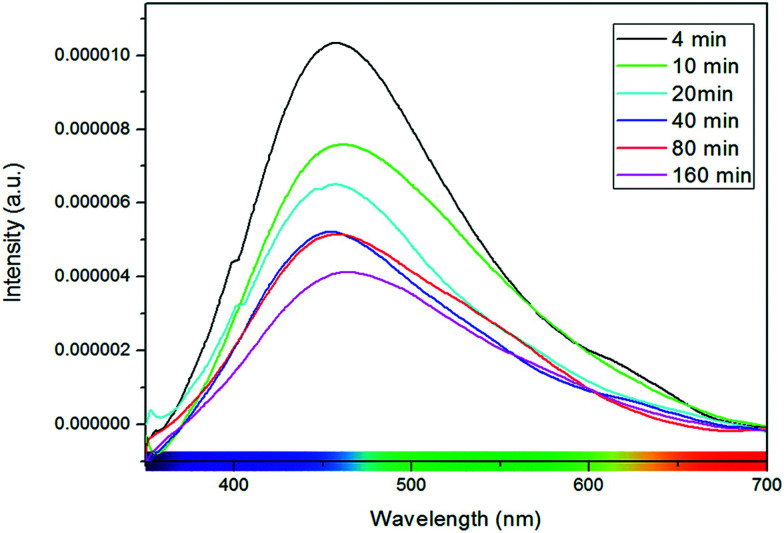
Photoluminescence spectra of STO nanoparticles with different synthesis times. Longer synthesis times correspond to larger particles and to lower photoluminescence intensities. Reprinted with permission from ref. 114. Copyright 2012 American Chemical Society.

Other factors commonly correlated with photoluminescence in STO are morphology and lattice disorder.^112–116^ Meng *et al.* found that photoluminescence intensity increased in STO nanoparticles and ascribed this phenomenon to interface and surface states, increasingly present in smaller nanoparticles.^116^ Subsequently, it was also reported by other groups that the intensity of room temperature photoluminescence of STO nanoparticles decreased with increasing synthesis time and thus particle size (*cf.*Fig. 14)^114,115^ or more generally with increasing lattice order.^117–119^ Pontes *et al.* further found that amorphous thin films show an intense room temperature photoluminescence peak, that is absent for crystalline films. They correlated these differences to lattice distortions due to varying coordinations of O–Ti structures, more precisely five-fold coordinated Ti–O_5_ structure elements.^120–122^

A more detailed overview of several of the mentioned photoluminescence effects is given by Crespillo *et al.*^123^ In addition to the studies presented here, literature is also available for a variety of further doping materials, structural modifications and treatment techniques, used to alter the photoluminescence behaviour of STO. Examples range from Ni nanocrystals integrated in STO,^124^ doping of Li/La pairs on Sr sites,^125^ doping of Eu^126^ to a combination of HF etching with Ar^+^ irradiation,^127^ or high energy proton irradiation.^128^

### High temperature effects

**Fig. 15:**
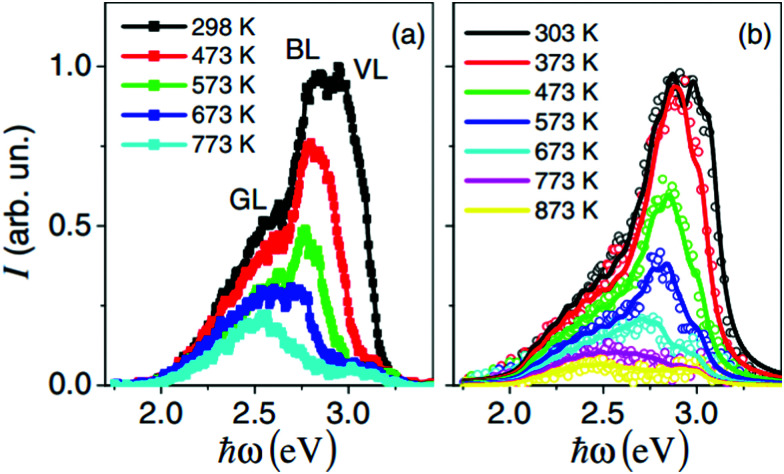
Photoluminescence spectrum with three separate luminescence bands (green – GL, blue – BL and violet – VL) of undoped STO at excitation energies of 2.2 mJ cm^−2^ (a) and 22 mJ cm^−2^ (b), recorded at temperatures between 298 and 873 K. Reprinted from ref. 111, with the permission of AIP Publishing.

Photoluminescence is usually reduced or even impeded at higher temperatures due to enhanced recombination. Therefore, photoluminescence is rarely studied at elevated temperatures. Rubano *et al.* performed a study at temperatures up to 600 °C and observed that photoluminescence effects are reduced when the temperature increases significantly above room temperature, however even at 600 °C they are still measureable.^111^ Furthermore, they found that the photoluminescence spectrum of undoped STO also undergoes a redshift with increasing temperature, possibly due to increased vibrational energy release (see Fig. 15).

We can summarize that the details of origin and dynamics of photoluminescence processes in STO are still far from being fully understood. However, it is clear, that not only intrinsic defects such as oxygen vacancies, but also impurities acting as trap levels in the STO band gap and deliberate doping, all play a substantial role in the question of color, intensity, decay dynamics and temperature dependence of the observed photoluminescence phenomena. We believe, that the research community would highly profit from a correlation of exact measurements of ionic defect types and concentrations in specific single crystals (on ppm-scale) and a corresponding systematic study of the origin of the different luminescence phenomena as this could present a sensible instrument for the identification of defects in STO and partly unveil remaining ambiguities in its defect chemistry.

## 5 Photovoltage

**Fig. 16:**
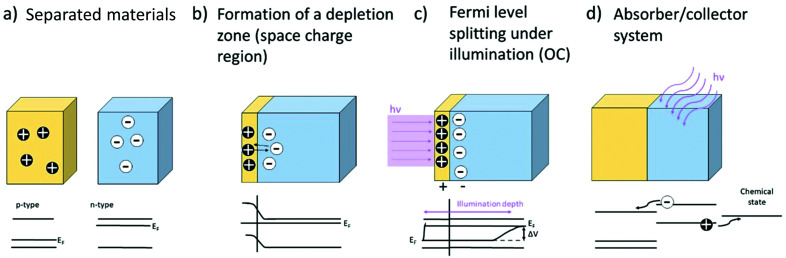
Schematic representation of the generation of a photovoltage at a heterojunction. A p-type and an n-type material are brought into contact (a), forming a space charge region with a built-in field (b). Under UV illumination and generation of electron/hole pairs, a splitting of the Fermi level occurs under open circuit conditions (c). Absorber/collector systems use semiconductors or Red-Ox systems to separate generated charge carriers in the resulting energy landscape (d).

Apart from light emission, photogenerated electron–hole pairs (see Fig. 3) can also lead to the formation of photovoltages. A photovoltage can be created whenever charge separation occurs subsequent to the charge carrier generation due to the characteristics of the present electronic band structures, *i.e.* when a band offset/band bending is present, *e.g.* at heterojunctions, surfaces/interfaces or pn-(pin-)junctions where, by equilibration of the Fermi levels, a space charge zone is formed. Here, the built-in field hinders the transfer of the respective majority charge carriers and prevents recombination. The lateral extent of this space charge region can vary for different materials and strongly depends on the charge carrier concentrations. Another way of creating and separating charge carriers is the use of an absorber, which can also be a semiconductor or even an organic dye. An absorber exhibits excitation levels in a certain wavelength spectrum and the absorption of light within this range leads to the generation of free charge carriers (*i.e.* electrons or holes). These can then subsequently be separated and partially transported into a (second) connected semiconductor. The overall principle for formation of photovoltages is depicted in Fig. 16. Above-band gap energy light generates charge carriers, which are then separated according to their charge, thereby creating a photovoltage.

**Fig. 17:**
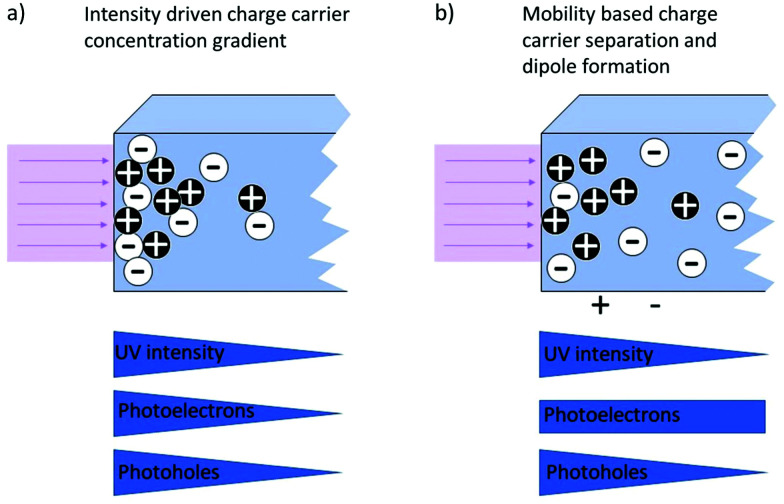
Schematic representation of the photo-Dember effect showing the intensity driven depth dependence of the generation of photoinduced charge carriers (a) resulting in a voltage due to differences in the mobilities of electrons and electron holes (b).

In addition, the photo-Dember effect can lead to a lateral or vertical photovoltage. According to the Lambert–Beer law, the intensity of light decreases with the depth, leading to a lower generation of photoinduced charge carriers in deeper regions of the semiconductor (see Fig. 16). A gradient of electron/hole concentration within the semiconductor is formed with a higher concentration of electron–hole pairs close to the surface. The photo-Dember effect then occurs based on the different mobilities of electrons and holes (both diffusing from their point of generation with their respective diffusion length) in a semiconductor, leading to a charge separation, given the diffusion lengths are different. Thereby a dipole and local voltages are created (see Fig. 17).

Photovoltaic effects in STO are important with regard to photocatalytic reactions^129^ and can be used for *e.g.* solar cells^130^ or photodetectors.^82,131,132^ The photoresponse of such systems is usually fast (at moderate and low temperatures) – owing to fast electronic processes – with rise times in the hundred ps range upon illumination with a laser pulse.^133^ However, recently, high temperature devices with additional slow time dependent processes have been reported with a voltage decay in the range of 10 to 100 s. This behaviour has been attributed to ionic effects under UV light.^14,134,135^ Therefore, we split the discussion into a lower temperature regime, in which primarily electron–hole generation and subsequent charge separation are the source of the voltage, and a higher temperature regime, in which a change in oxygen stoichiometry and oxygen transport under illumination have to be considered.

### Low temperature effects

Starting with undoped STO surfaces, the wavelength-dependent absorption of STO single crystals has been reported,^133^ showing a slight increase from 300 to 385 nm and a sharp drop in absorption at 385 nm. The photovoltage for these single crystals at 355 nm exhibits ultra-fast photoresponse, with a rise time of 130 ps and a full width at half maximum time of 230 ps with peak photovoltages of up to 52 mV upon UV laser irradiation.^133^ In addition, a dependence of the photovoltage on the tilt angle of miscut STO single crystals is reported,^133^ showing a maximum in photovoltage at a tilt angle of approx. 20° with regard to the (100) direction. The photovoltage was measured upon irradiation of an area between two painted indium electrodes which were ∼1 mm apart and kept in the dark. Similar results were obtained for Nb doped STO single crystals.^136^ The above mentioned wavelength dependence has been actively employed for UV-sensitive, but visible blind photodetectors with a lateral electrode design on STO single crystals with a reported cutoff wavelength of 390 nm.^131^ Regarding the origin of the STO single crystal based photovoltages, a model considering contributions from photoelectronic processes and the Seebeck effect^133^ and additionally, a surface-barrier model with 
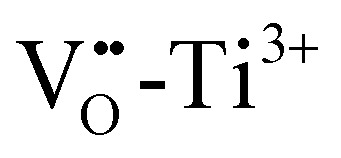
 dipole centers oriented in the field have been suggested.^137^ It has also been shown that the photoresponse of STO single crystals depends on the electrode material, showing differences in photocurrent, photovoltage and rise time using Ag, Pt or Ni electrodes.^138,139^ Upon irradiation with a 15 ps laser pulse with a wavelength of 355 nm, photovoltages of approx. 0.8 V, 1.0 V and 1.1 V with rise times of 301.5, 394.4 and 360.9 ps and full width at half-maximum times of 537.2, 966.9 and 576.5 ps for Pt, Ni and Ag, respectively, are reported.^138^ A similar experiment was performed by Jin *et al.* (Au and Pt), yielding slightly higher photovoltages for Pt in the temperature range from 80 to 300 K due to the difference in work function and, thus, in Schottky barrier height.^139^

Photogenerated charge carriers in undoped STO thin films on STO single crystals can lead to substantially different effects, as the occurring photovoltage can be tailored *via* the oxygen vacancy concentration.^140^ By applying an electric field, oxygen vacancies move towards the negative electrode and affect the measured photovoltage *via* band bending. Consequently, two reversible photovoltaic states, even with a change in the sign of the photovoltage, have been reported.^140^ STO thin film based photodetectors operated at 10 V show a photovoltage of approximately 0.25 V^82^ with a rise time of 330 ps and a full width half maximum of 700 ps when irradiated with a 355 nm laser pulse of 25 ps. Bias effects on the photovoltage have also been demonstrated for STO single crystals operated at a 10 V bias, which could be used as photodetectors with photovoltages of 2.4 V for one cell and up to 8.1 V for four cells in series when irradiated with a 375 nm laser with 10 mW cm^−2^.^141^ However, using fewer cells (*e.g.* just one cell) results in a faster photoresponse in the range of hundreds of picoseconds.^141^

Beyond single crystals and homoepitaxial thin film growth, STO has been frequently employed in heterojunctions (*e.g.* Schottky or p–n junctions) for the generation of photovoltages. For more information on dye sensitized solar cells, the reader is referred to the review by Suzuki *et al.*^142^ Despite the properties of heterojunctions cannot be solely ascribed to one constituent, in the present review, STO heterojunctions with different top layers are discussed, ranging from organic or metallic coatings to inorganic and oxide top layers, where throughout, photovoltages of several 100 mV have been observed. For instance, Yamaura *et al.* reported photovoltages of up to 0.7 V in poly(3,4-ethylenedioxythiophene) PEDOT/STO junctions.^143,144^ In addition, a multilayer consisting of indium tin oxide (ITO), lead zirconium titanate (PZT), STO and GaAs yields a photovoltage of up to 400 mV in simulated sunlight.^145^ Regarding the probably best-known STO-based interface LAO/STO,^146^ a one unit cell thick layer of LAO on STO has been investigated by Liang *et al.*^147^ A dependence of the photovoltage on the work function of the metal top electrode (Ag, Au, Pt) is found, increasing from 0.2 V for silver to 0.4 V for Pt. When lowering the energy of the light source from 6.7 eV to below the band gap of LAO, however, the photovoltage decreases from 0.3 V to below 0.1 V for 3.4 eV and solar light. A residual polar field in the ultrathin LAO thin film is suggested, which influences the photovoltage.^147^ The photovoltage at the LAO/STO interface is therefore mostly influenced by LAO.

Beyreuther *et al.* investigated manganite/STO heterojunctions (for a more detailed report on such heterojunctions, please refer to a review article by Luo *et al.*^148^) using surface photovoltage spectroscopy (SPV). They reported mainly STO related states to be responsible for observed photovoltages (*i.e.* optimization should focus on STO),^149^ however, specific thin film related states were detected in accordance with another work.^150^ Such an optimization of undoped STO can be achieved *via* self doping, *i.e.* the introduction of oxygen vacancies acting as donor dopant, yielding n-type STO. Different cells using self doped n-type STO have been investigated, *e.g.* STO/Si,^151,152^ STO/GaAs^153^ and STO/Pt.^18^ For a p–n junction of p-type Si and n-type SrTiO_3_ (*via* oxygen vacancies), photovoltages above 100 mV are observed.^151,152^ In the work of Wen *et al.*, an n-type STO thin film is deposited on a p-Si substrate.^152^ There, visible light can pass through the STO thin film (band gap 3.2 eV) and leads to the generation of electron–hole pairs in the p-Si. The built-in field leads to charge separation by moving the electrons to the n-type STO. In contrast, when using UV light, the photoinduced charge carriers are formed in the STO thin film. Then again, charge separation happens at the interface, leading to a movement of the holes to the p-Si. However, the recombination rate of the photoinduced charge carriers is higher in STO and higher photovoltages were obtained for visible light (632.8 nm) than for UV light (355 nm).^152^ Jin *et al.* investigated STO single crystals which were vacuum annealed at different temperatures with Pt top electrodes.^18^ The “as received” sample yielded the highest photovoltage in the study with 1.1 V at 60 K, decreasing down to approx. 200 mV at 300 K. In comparison, samples initially annealed at 650 in vacuum exhibited a photovoltage below 100 mV at 60 K. However, between 200 and 250 K the measured photovoltage peaks at approx. 0.5 V. This demonstrates that reducing STO is an effective way of tailoring the temperature dependence of the photoresponse of STO-based systems.

Solid solutions and composite materials based on STO powders also show promising photovoltaic properties. For example, an STO/TiO_2_ composite exhibits an increased photoresponse compared to both isolated materials, STO and TiO_2_.^154^ This effect is attributed to the band structure of the STO/TiO_2_ interface enabling an enhanced charge carrier separation and thus leading to a lower recombination rate of the photoinduced charge carriers.^154^ In addition, photoelectrical properties of STO and BiFeO_3_ (BFO) solid solutions have been investigated,^155^ yielding photovoltages >1 V.

Owing to its n-type nature, pn-junctions are often investigated based on Nb:STO, frequently using p-type (lanthanum) manganites. An overview of the manganite/Nb:STO heterojunctions is shown in Table 1, and the results of other top layer materials on Nb:STO are shown in Table 2. Photovoltages up to 1 V have been reported, but comparison between values obtained in different studies is often difficult due to different excitation wavelengths (ranging from UV to VIS light), light intensities, measurement modes (vertical *vs.* lateral), and temperatures.

**Table 1:** Parameters for manganite/Nb:STO based heterojunctions

Material on Nb:STO	*T* (K)	*λ* (nm)	Energy	*V*_OC_ range (mV)	*V*_OC_ (mV) at RT	Ref.
Doped PrMnO_3_	20–300	365, 473, 532	0.3–70 mW mm^−2^	0.2–20	5	156–158
Doped LaMnO_3_	17–390	210–660	0.3–15 mW cm^−2^ if specified	0.15–1000	15–1000	156 and 159–172
Alkali doped LaMnO_3_	80–300	248, 473	25–500 mW cm^−2^	50–580	300	173–175
Other manganites	80–300	365, 460, 660	2.6 mW mm^−2^ if specified	2.2–345	345	176 and 177

**Table 2:** Parameters for Nb:STO based heterojunctions

Material on Nb:STO	*T* (K)	*λ* (nm)	Energy	*V*_OC_ range (mV)	*V*_OC_ (mV) at RT	Ref.
YBa_2_Cu_3_O_7_	40–350	355 – solar light	0.5–6 mW mm^−2^	0.1–1040	780–1040	180–183
Perovskite oxides	80–300	248, 266, 532	2.12–14.86 mW mm^−2^	100–400	100	184–186
Other metal oxides	RT	248, 308, solar light	Unspecified	3.3–565	565	178 and 187–189

**Fig. 18:**
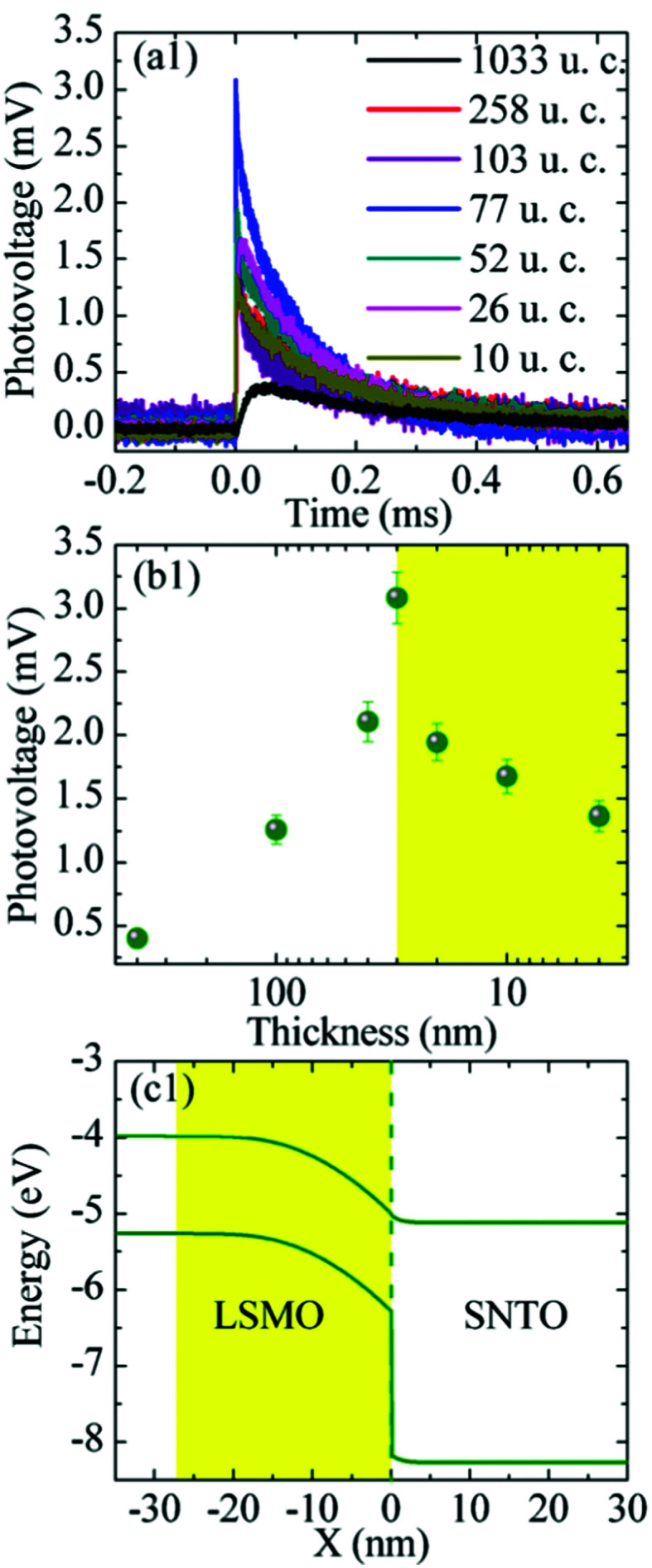
(a and b) Thickness dependence of the photovoltage developing at the junction of a La_0.9_Sr_0.1_MnO_3_ thin film on Nb doped SrTiO_3_ (energy landscape shown in (c). A maximum is found for approximately 30 nm which is the width of the depletion region. Reprinted from ref. 161, with the permission of AIP Publishing.

In cells using manganite top layers (see Table 1), the importance of the thin film has often been emphasized,^160,166,173–175^ highlighting the effect of a metal/insulator transition. For instance, the maximum photovoltage for La_0.9_Li_0.1_MnO_3_ is reported at 240 K, close to the transition temperature of the material. For La_0.7_Ce_0.3_MnO_3_ thin films, also the importance of magnetic properties has been shown. Introducing oxygen vacancies weakens the ferromagnetic ordering and a lower photovoltage is observed.^169^ The use of external magnetic fields may further affect the photovoltage. Magnetic suppression has been reported for Ca and Sr codoped praseodymium manganite.^158^ Wu *et al.* investigated the temperature dependence of Nb:STO based PV cells with hafnium doped lanthanum manganite thin films as a top layer.^164^ While magnetoresistance of the thin film could be demonstrated, no dependence of the photovoltage on the magnetic state was found. Higher photovoltages found at lower temperatures are attributed to a thicker depletion region or a higher diffusion voltage of the built-in field at lower temperatures. Due to the importance of the depletion region, also the thickness of the manganite thin films is affecting the photovoltage of STO.^161,171,178^ For La_1−*x*_Sr_*x*_MnO_3_ (LSM), an optimum thickness of the length of the depletion zone in LSM was found,^161,171^ see Fig. 18. Thicker films enhance recombination, whereas in thinner films, the built-in field is weakened. For the LSM/Nb:STO interface, also an insulating interlayer was shown to enhance the photovoltage.^162^ Another way of influencing the photovoltage of systems with manganite top layers is *via* resistive switching, *e.g.* in Au/Pr_0.7_Ca_0.3_MnO_3_/Nb:STO/Au and Au/La_0.7_Ca_0.3_MnO_3_/Nb:STO/Au.^156^ Similarly, a change in photovoltage was observed in Au/Nb:STO systems with different resistive states of the junction, ranging from 0.003 mV at 70 MΩ to 57.6 mV at 900 MΩ.^179^

Apart from manganites, different other oxides have been used as top layers in Nb:STO based photovoltaic cells (see Table 2). Here, the YBa_2_Cu_3_O_7_ (YBCO)/Nb:STO heterojunction^180,182,183^ overall yields high photovoltages, *e.g.* 1.04 V in solar light. Hao *et al.* investigated the use of different irradiation wavelengths and intensities, as well as the effect of different temperatures on YBCO/Nb:STO interfaces, observing higher photovoltages at low temperatures, high light intensities and lower wavelengths.^181^ Indeed, the temperature dependence of the photovoltage shows a kink at 100–120 K. This temperature is attributed to the phase transition from cubic to tetragonal STO^181^ and the band gap increases from 3.2 eV at 4.2 K to 3.23 eV near the phase transition and decreases with temperature after the phase transition, thus explaining the kink in the recorded photovoltage curve.^181^ In contrast, the superconducting transition of YBCO at 90 K does not result in any significant feature in the temperature dependence of the photovoltage.^181^ Furthermore, the YBCO/Nb:STO interface can also be affected by oxygen annealing. Showing no photovoltaic effect at annealing oxygen pressures of 10^−5^ Pa or lower, the photovoltage increases with the oxygen annealing pressure up to a maximum of approximately 0.7 V at 10^2^ Pa and, after a minimum at 10^3^ Pa, a stable plateau at 0.5 V in the 10^4^ Pa region is reached. The oxygen partial pressure dependence of the photovoltage correlates with the semiconductor–metal transition, changing the interface from a p–n junction to a Schottky junction.^183^ Another example for the effect of oxygen annealing (although at low temperatures) was given by Wang *et al.* for another metal oxide as a top layer, namely for NdNiO_3_(NNO)/Nb:STO heterojunctions. Here, oxygen annealing leads to a dramatic change in the resistance of the NNO thin films, while comparable photovoltages in the 0.18 to 0.22 V range were obtained.^189^

Regarding the effect of the Nb:STO substrate, the impact of different Nb doping concentrations on the photovoltage was investigated,^178^ showing an increasing photovoltage for higher Nb doping concentrations. This is explained by the stronger built-in fields that result for higher donor concentrations in the n-type conductor of a p–n junction.^178^ The angular dependence in the photoresponse was observed in miscut Nb:STO single crystals^136^ and also for LCM layers on top of miscut Nb:STO single crystals.^190^ Additionally, a thickness dependence in the peak photovoltage of miscut Nb:STO single crystals upon illumination with 248 nm laser was found, yielding 180 μm as the optimal thickness. The vertical photovoltage was compared with the lateral photovoltage due to the photo-Dember effect^185,191^ and higher voltages with faster relaxation times were found in the vertical case. In both cases, the transport of photoinduced charge carriers is supposed to take place mainly in the Nb:STO substrate.

### High temperature effects

**Fig. 19:**
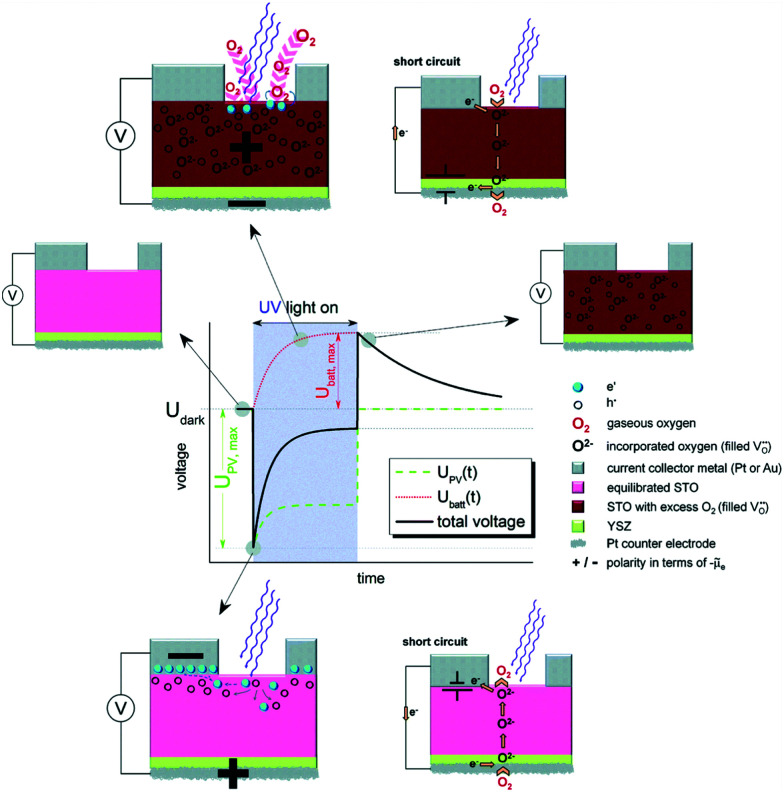
Schematic voltage *vs.* time measurement of an STO single crystal with the corresponding stoichiometric states before, during and after UV illumination at elevated temperatures (300–400 °C), highlighting the ionic character of the time dependent behaviour. Reproduced from ref. 14 with permission from the Royal Society of Chemistry.

While in the temperature regime below 300 K, usually higher photovoltages were found at very low temperatures,^181^ surprisingly high photovoltages can also be observed at higher temperatures. Brunauer *et al.* demonstrated that La_0.8_Sr_0.2_CrO_3_/STO heterojunctions coupled with a solid oxide fuel cell can be operated at temperatures between 400 and 500 °C with photovoltages close to 1.0 V.^23^ Such cells can also be combined with a solid state electrochemical cell and the photovoltage from the solar cell can be used to pump oxygen through the electrochemical cell. Thus, in principle, this combination could be used for photopowered water splitting and consequently for solar hydrogen production. The high temperature photovoltage depends on the top electrode (on the illuminated side). Different perovskite-type oxides and metals have been used as top layers, *e.g.* La_1−*x*_Sr_*x*_Cr_1−*y*_Mn_*y*_O_3_ and Au, respectively, with overall high photovoltages of up to above 1.1 V.^135^ A time dependent behaviour upon switching the UV light on or off is observed, however, the time scales here are not in the ps to μs range as they are for the low temperature counterparts, but rather in the couple of minutes range. These slow processes are – in fact – not attributed to electronic processes, but to ionic changes, particularly to a change in oxygen stoichiometry upon illumination *via* faster oxygen incorporation.^13,22^ When operating such a solid oxide solar cell in short circuit mode, the photocurrent also exhibits a time dependent behaviour, leading to increasing photocurrents under illumination. Here, it has to be considered that the electrodes are ionically blocking and that stoichiometry polarization effects may lead to more conductive regions.^192^ The decrease of the STO bulk resistance thus leads to higher photocurrents, self-enhancing the power of such a high temperature solar cell. In the work of Walch *et al.*,^14^ STO single crystals were investigated between 400 to 500 °C in air using different metal current collectors and also using YSZ bottom layers. Again, a time dependent behaviour upon switching the UV light on or off was found (see Fig. 19).

The change in oxygen incorporation upon illumination influences the photovoltaic voltage, as demonstrated in the low temperature counterpart.^183^ Upon switching off the UV light, the photovoltaic voltage goes down to zero almost immediately. However, the changed oxygen stoichiometry persists and – without illumination – is no longer in equilibrium with the gas phase. A Nernst-like (battery-type) voltage results due to the difference in the oxygen chemical potential. The excess oxygen is then slowly released, thereby leading to a slow decrease in this battery type voltage, until the equilibrium with the gas phase is reached. Different metals have been used as a current collector to change the Schottky contact, with Au reaching higher voltages than Pt, in contrast to the results of low temperature measurements.^139^

Also, Nb:STO based heterojunctions were operated at high temperatures and the temperature dependence of La_2/3_Ca_1/3_MnO_3_ (LCMO)/Nb:STO heterojunctions was studied in the temperature range from 293 to 723 K.^193^ At first, a decrease in the peak photovoltage upon UV laser illumination was observed from 142 mV at 293 K to 48.7 mV at 523 K. When increasing the temperature further, the photovoltage increased up to 118 mV at 723 K. As the Schottky barrier height is not expected to change with temperature, only the barrier width might change, which was shown to be tightly correlated with the migration of oxygen vacancies at higher temperatures.^193^ Ni *et al.* propose the following explanation: With increasing temperature, the recombination of UV induced charge carriers is enhanced and the lifetime of these charge carriers is reduced, causing the decrease in voltage from 293 to 473 K.^193^ Also, oxygen migration at higher temperatures leads to a change in the oxygen stoichiometry at the interface and, thus, to a change in barrier width. Tunnelling of electrons through the interface barrier is enhanced, which leads to the increase in photovoltage at higher temperatures.

The ITO/Nb:STO junction was studied at 873 K under UV illumination (365 nm) with an irradiation intensity of 261.2 mW cm^−2^ under different oxygen partial pressures.^130^ At 1 bar *p*(O_2_), a photovoltage of 123 mV was observed, which decreases with lower oxygen partial pressures, *e.g.* 30 mV at 10^−4^ bar. Oxygen partial pressure changes are suspected to change the barrier height, thereby influencing the photovoltage.^130^

In conclusion, with regard to photovoltages measured at low temperatures (*i.e.* below 300 K), a large variety of different top layers on Nb:STO ranging from different manganites to yttrium barium copper oxide to various other oxides has been investigated. Here, especially among manganite thin films, a lot of research has been carried out, but a more systematic approach would be helpful for the field of oxide photovoltaics and to direct and to focus research efforts. For undoped SrTiO_3_, different factors affect the photovoltage apart from the top layer, including temperature, annealing atmosphere, film thickness, magnetic film properties, and miscut angle. At higher temperatures, there are fewer publications on the photovoltaic properties of SrTiO_3_ based cells. Interestingly, more data is available on undoped SrTiO_3_ here, often with surprisingly high photovoltages of up to 1.1 V. Here, ionic contributions also take place during measurement, inducing processes on the time scale of minutes or even hours, compared to the ns or ms transient behaviour of low temperature counterparts.

## 6 Photochromism

STO has been known to change its color under UV illumination, a phenomenon commonly known as photochromism. In general, this is due to newly populated energy levels in the band gap and accompanying changes of the absorbance and thus the color of the material. Again, this effect may be observed at low as well as at high temperatures and is always correlated with specific defects and oxidation states.

### Low temperature effects

In the low temperature range between 1.8 K and room temperature, color centers are usually formed under irradiation with light in the range of 390 and 485 nm.^194–196^ These color center formation processes are reversible, and bleaching can be achieved thermally or through illumination with light with a wavelength of 530–800 nm. In Fe:STO under oxidizing conditions, the Fe dopant^197^ exhibits the oxidation states Fe^3+^ and Fe^4+^ on the Ti^4+^ site. To compensate negative charge caused by Fe^3+^, 
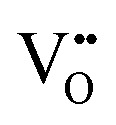
 and 
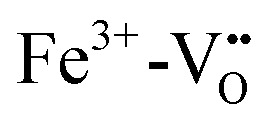
 clusters are formed. Under irradiation, the formation of Fe^4+^ and surprisingly even Fe^5+^ is reported,^197^ both at the expense of Fe^3+^. Subsequently, such specimens exhibit photochromic absorption in the visible light range due to a charge transfer either from the Fe^4+^ state to another Fe^4+^ state (internal d–d transition)^197^ or from electron excitation from the valence band (mainly consisting out of O^2−^ states) into the Fe^4+^ ^197,198^ or Fe^5+ ^^198^ state, creating finally Fe^3+^ for both cases.^198^ Such electron excitations can also be caused thermally or due to long wavelength irradiation and can be used to purposely bleach the specimens again.

**Fig. 20:**
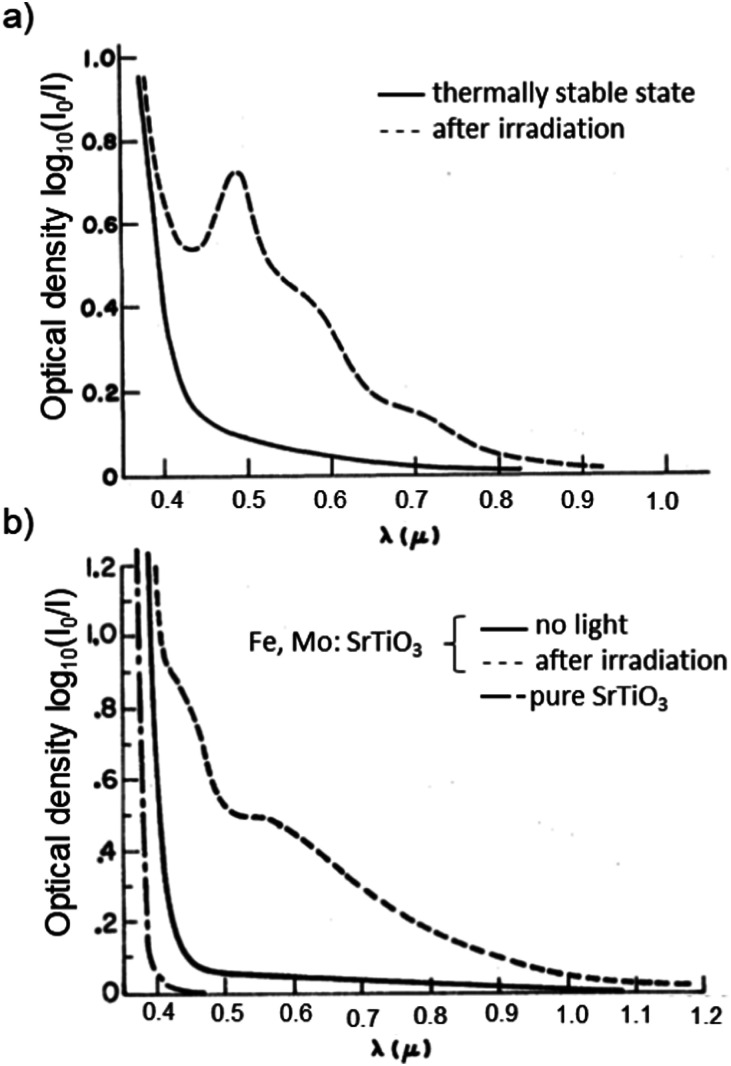
Absorption spectra of (a) Ni:STO and (b) Fe,Mo:STO before and after irradiation with light in the range of 390 to 430 nm at 77 K. Illumination leads to a photochromic effect in both materials, broad absorption bands evolve in the visible light range. Adapted with permission from ref. 199. Copyright 1971 by the American Physical Society.

In Fe and Mo codoped STO, Fe^3+^ is compensated by the formation of Mo^6+^ and during illumination, three different processes can cause a photochromic effect. Firstly, an electron can be excited from the Fe^3+^ level to the conduction band. Secondly, electrons can be transferred from the valence band into the conduction band with the electron holes then being trapped by Fe^3+^ and electrons potentially being trapped by 
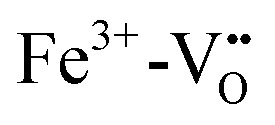
 clusters, forming either Fe^4+^ or 
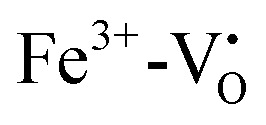
, respectively. Thirdly, an electron may be excited from the valence band into the Mo^6+^ state, causing the formation of Mo^5+^ with the electron hole again being trapped by an Fe species.^199^ In conclusion, Fe^3+^ may be oxidized to Fe^4+^ and Mo^6+^ may be reduced to Mo^5+^ due to irradiation with light in the range of 390 to 430 nm at 77 K.^195,196^ In Fig. 20(b), the absorption spectra before and after illumination are shown at 77 K.^199^ When codoped with Mo and Ni, Ni^2+^ is oxidized to Ni^3+^ and Mo^6+^ is reduced to Mo^5+^ during irradiation with light in the range of 390–440 nm.^196^ Both codoping systems can be bleached thermally or through long wavelength illumination.^195,196^

In monodoped Ni:STO, Ni species can act as both electron donors and electron acceptors. Although experiments have only shown the presence of Ni^2+^ in untreated samples before illumination with 390 to 430 nm radiation at 77 K, it is assumed in literature that 
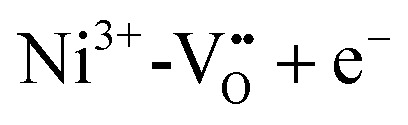
 clusters are undetected but present additionally. After irradiation, the sample exhibits a smeared-out absorption edge attributed to an acceptor type charge transfer from the valence band to the 
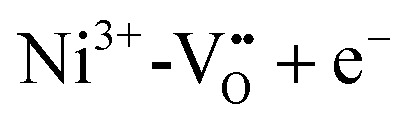
 cluster, forming 
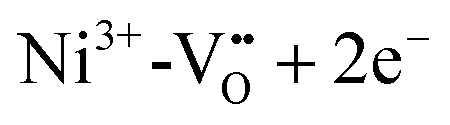
 or a donor type charge transfer resulting in the formation of Ni^3+^ from Ni^2+^. Additionally, two further donor type absorption bands at 575 and 480 nm are present. At these wavelengths 
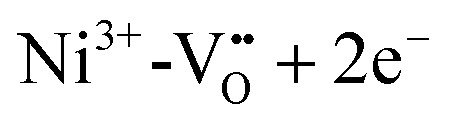
 is changed back to 
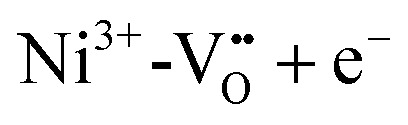
 and the excited electrons are transferred either to the conduction band or most probably to an unstable state, from which they undergo a relaxation process. At 520 nm Ni^3+^ is oxidized to Ni^4+^ and the excited electron is captured by another Ni^3+^ center or a 
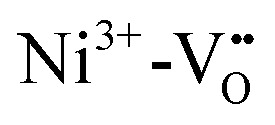
 cluster forming 
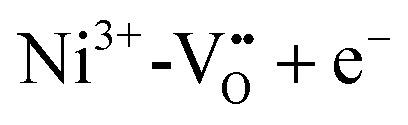
. An absorption spectrum of Ni:STO is shown in Fig. 20(a). The described processes were examined using EPR and/or light absorption techniques.^199,200^

Other examples for dopants causing a photochromic effect in STO below room temperature are Co, Cr, and V.^199^ Since the processes behind their photochromic effects are similar, we will not discuss these materials in more detail.

### High temperature effects

**Fig. 21:**
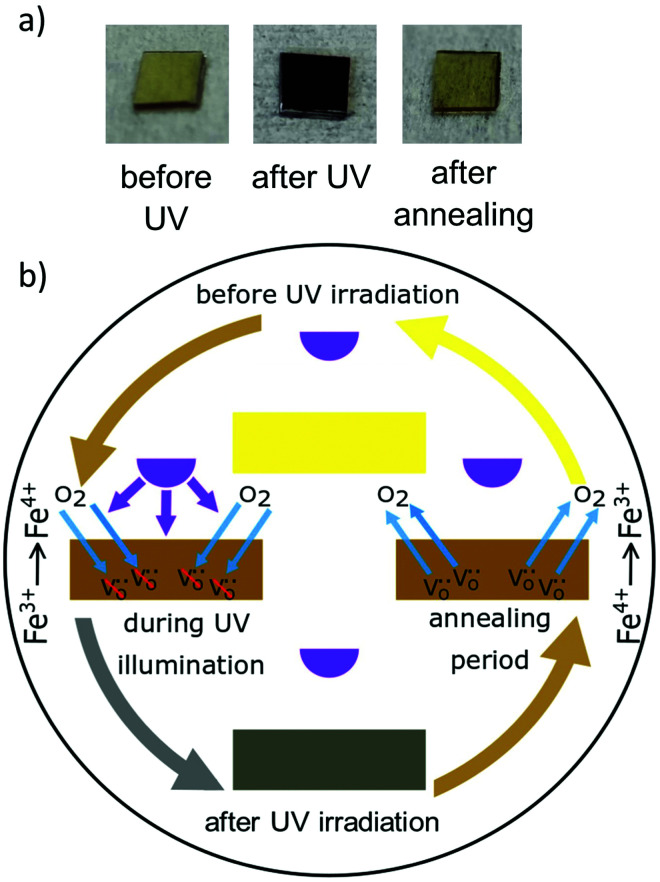
(a) Photographs of Fe-doped STO single crystals, before UV irradiation, shortly after UV exposure, and after annealing at 700 for 12 h in air. (b) Schematic illustration of high temperature photochromism of Fe doped STO. Oxygen incorporation leads to an oxidation of Fe^3+^ to Fe^4+^ during UV irradiation. The initial state can be re-established by annealing after the UV light is turned off. Adapted from ref. 22, with the permission of John Wiley and Sons.

In the high temperature range only Fe:STO has so far been reported to show photochromism. Above 350 °C, a color change from brownish transparent to dark brown/black can be observed after above-band gap irradiation (compare Fig. 21^201^). The process behind the color change at such temperatures is again different to the processes responsible for the low temperature photochromism. Here, UV illumination leads to an enhanced oxygen incorporation^13^ and therefore to the formation of Fe^4+^. Fe^4+^ exhibits broad absorption bands in the range of 440 and 590 nm.^201^ The change in the oxygen vacancy concentration is accompanied by an increase of the conductivity. The initial state is restored within three days at 350 °C after the UV source is turned off. Annealing at higher temperatures (*e.g.* 700 °C) leads to an enhanced oxygen diffusion and release in Fe:STO single crystals, and therefore shortens the time for the re-establishment of the initial color. Fig. 21 schematically displays the color changes due to UV irradiation and annealing in air. If the specimen is quenched to room temperature during or instantly after the UV light is turned off, the color change can be preserved nearly permanently, since the kinetics for oxygen diffusion and surface exchange reactions become extremely slow.^201^

## 7 Photocatalysis

In 1972, Fujishima and Honda discovered the ability of TiO_2_ and Pt to function as photoanode and cathode to facilitate water splitting under UV light irradiation.^202^ Since their experiments, related semiconductors such as ZnO,^203–206^ SnO_2_,^207–211^ Fe_2_O_3_,^212–215^ and STO attracted a lot of interest as photoanodes and were studied extensively throughout the last decades. Among others, STO became one of the best investigated compounds and applications in the field of photocatalytic overall water splitting,^216–225^ applications for H_2_ evolution,^226–237^ CO_2_ conversion,^238–240^ inorganic/organic pollutant, NO_*x*_, SO_*x*_ and dye degradation,^233,241–254^ N_2_ fixation,^255^ and bacteria inactivation^256^ were also reported.

Operation at ambient conditions is most important for technological processes and therefore also preferred in research. Regardless of operation conditions, electrons have to be excited from the valence band to the conduction band by illumination in order to utilize solar energy for photocatalytic processes. Subsequently, the photogenerated electrons and holes need to be separated, and the charge carriers have to be transferred to active sites (either the surface of STO or of loaded cocatalysts), where oxidation or reduction processes take place. Hence, to understand the photocatalytic properties of STO, detailed knowledge of the band structure of STO is necessary.

Compared to TiO_2_, undoped STO exhibits a conduction band closer to the vacuum level and a higher electron mobility. It is innately capable of photocatalytic water splitting, since its electrons/holes in the conduction/valence band are able to reduce/oxidize water, respectively. The valence band consists mainly of O_2p_ orbitals and its upper edge is positioned at 2.59 eV *vs.* NHE.^52^ The lower edge of the conduction band is located at −0.61 eV *vs.* NHE^52^ consisting predominantly of Ti 3d states.^257^

Since only a small part of the solar radiation on the earths surface has a wavelength below ∼390 nm^258^ and therefore is capable to excite electrons from the valence to the conduction band in STO at room temperature, it is desired to expand the range of usable wavelengths. For improving the overall photocatalytic activity of STO, the photogenerated charge carrier separation, charge transport and finally the reduction/oxidation reactions also have to be optimized. For these complex tasks, various approaches and techniques have been developed and described in the last decades. Several review articles provide an overview of these aspects for STO or related materials.^52,258–261^ In the following chapter, existing STO-based photocatalytic systems will be described and the most important strategies will be summarized to improve catalytic activity either by doping, the formation of heterojunctions, or further techniques (*e.g.* introducing semipermeable protective layers or carboxyl groups at the surface). Again, the effects described in this chapter are often not solely based on SrTiO_3_ but on heterojunctions with different materials.

### 7.1 Doping

The intentional introduction of impurity atoms changes the electronic structure of the host material and consequently, doping is regularly used to tailor the band structure with the aim of enhancing certain properties such as conductivity and photocatalytic activity. To enhance the photocatalytic properties of STO, various dopants *e.g.* Na,^262,263^ Li;^262,263^ K^262,263^ Rb,^262,263^ Cs,^262,263^ Mg,^262,263^ In,^262,263^ Ga,^262,263^ Ir,^264,265^ Nb,^266–268^ Rb,^263^ Fe,^241,254^ Cr,^226,236,269–274^ Sc,^275^ Ni,^266,276–279^ La,^236,263,274,276–282^ Rh,^281–285^ Al,^240,262,263,286–288^ Sb,^219,284,289^ Ta,^226,266,269,279^ Co,^287^ Cu,^287^ Mn,^290^ B,^272,291^ N,^232,280,292–296^ S,^293,295,297,298^ C,^267,294,297,298^ P^291^ or F^251,291^ were reported. Since doping with only one element may lead to the formation of very active recombination centers, and other disadvantages, codoping with either two cations, two anions or one cation and one anion became popular.^236,289^

#### Cationic monodoping

In STO, doping with Al (Al:STO) leads to the incorporation of Al^3+^ on the Ti^4+^ site and to a shift of the absorption edge to approx. 390 nm. In combination with Ag loading on the surface, a highly selective CO_2_ conversion towards CO was achieved. The best results were achieved with 4 mol% Al doping.^240^ Other systems consisting of Al:STO and cocatalyst heterojunctions are used for hydrogen evolution reactions^286^ or overall water splitting^288,299^ with external quantum efficiencies of up to astonishing 96% at 350 and 360 nm.^299^

Cr doped STO nanoparticles can be used for NO_*x*_ degradation and photocatalytic activity could be found under red light irradiation. Thereby a charge transfer from Cr^3+^ to the Ti_3d_^4+^ orbital in the conduction band is responsible for the generation of ˙O_2_^−^ and subsequently the formation of ˙OOH. Due to the reaction of ˙OOH, molecular oxygen, water and NO, HNO_2_ and HNO_3_ are formed.^271^ In the degradation of gaseous isopropyl alcohol, Cr doping is also used to expand the absorption to the visible range and consequently improves the photoactivity of the Ag_3_PO_4_/Cr:STO^300^ and g-C_3_N_4_/Cr:STO^273^ heterojunctions. Even though Cr^3+^ enhances the efficiency of water splitting tremendously, in monodoped Cr:STO, also Cr^6+^ is formed which is known to be a recombination center for photogenerated electrons and holes.^236^

Fe:STO may be used for organic pollutant degradation.^241,254^ For Rhodamine B (RhB) degradation, it is suggested that Fe:STO shows high visible light photocatalytic activity, due to Ti^4+^–O^2−^–Fe^2+^ linkages at and nearest to the surface. However, Fe energy states in the band gap can also act as electronic traps.

Further, Mn doped STO can be used for water purification, where Mn^4+^ and Mn^3+^ coexist on the Ti^4+^ site and expand the photocatalytic activity to the visible light range,^290^ an effect that was also shown for Ru, Rh and Ir doped STO loaded with Pt as cocatalyst.^301^ In recent years, Rh doped STO became the basis of Z-scheme photocatalytic systems for water splitting under visible light, see also Section 7.3.^216,261,302–305^ Another application for Rh doped STO is the purification of wastewater *e.g.* by effectively killing *E. coli* bacteria under visible light. In this process, Rh^3+^ is present in the bulk as the photoactive substance and Rh^4+^ can be found at the surface.^256^ In the presence of only Rh^4+^ in STO, phages can be selectively inactivated by not harming *E. coli* bacteria at the same time.^306^

#### Cationic codoping

As mentioned above, single element doping can lead to the formation of recombination centers for photogenerated electron–hole pairs. Codoping is one way to overcome this problem. Ni doping of STO leads to the formation of Ni^2+^ and Ni^3+^. Ni^2+^ introduces new energy levels in the band gap of STO and facilitates hydrogen evolution in the visible light range. Ni^3+^ on the other hand is believed to trap photogenerated electrons.^266^ The introduction of La^3+^ ^276–279^ on the Sr^2+^ site or Ta^5+^ or Nb^5+^ ^266^ on the Ti^4+^ site reduces the concentration of Ni^3+^ in favour of Ni^2+^ due to charge balance reasons.^266,279^ The same principle is utilized in the case of Cr doping. Cr^3+^ is desired and Cr^6+^ a well-known recombination center, whose formation can be prevented by codoping with La,^274,307^ Nb,^269^ Sb^272^ or Ta.^226,269,308,309^ Rh doping is more complex, since for some applications Rh^3+^ is favourable and for others Rh^4+^. For example, codoping with La^281,282^ or Sb^219,284^ leads to the formation of mainly Rh^3+^ and highly effective surfaces for hydrogen evolution.^282^

#### Anionic monodoping

**Fig. 22:**
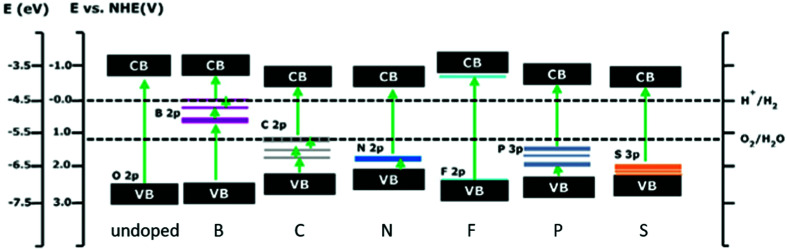
Calculated valence band and conduction band positions of undoped and non-metal doped STO. Additionally, the states, introduced by anionic doping, are displayed. Doping always amounts to 4.167 at%. Reprinted from ref. 291, with permission from Elsevier.

For replacing oxygen in the lattice, fewer elements are available than for cationic doping. Since S, N, P and C exhibit higher p-orbital energies than oxygen, doping with these elements should shift the top of the valence band upwards and as a consequence, narrow the band gap.^310^ However, all 2p states of the listed dopants except those of S show no strong interactions with the O_2p_ orbitals and therefore cannot influence the valence band substantially. The band gap narrowing is either very small for N^291,294,310^ or in the case of P doping quasi non-existent^291^ and only localized states above the top of the valence band are introduced.^291,294,311^ N_2p_ states are quite close to the top of the STO valence band, while the impurity states introduced by C and P are located unfavourably mid-gap.^291^ On the other hand, S_2p_ states mix with the O_2p_ states, shifting the top edge of the valence band and effectively narrowing the band gap.^291,311^ In Fig. 22, calculated band positions and impurity states, introduced by anionic doping, are displayed.

#### Anionic codoping

Often, anionic codoping leads to more stable compounds than monodoping.^310^ DFT calculations suggest that codoping of N and S causes synergistic effects and a strong band gap narrowing, which induces a good visible light absorption and may facilitate the separation of photogenerated electrons and holes.^295^ Codoping with C and S results in a mixture of O_2p_, C_2s_ and S_3s_ orbitals as top of the valence band. The conduction band is composed of Ti_3d_, C_2p_ and S_3p_ orbitals.^298^ Thiourea (CH_4_N_2_S) is claimed to not only be a good codoping source for C,S:STO but also for N,S:STO photocatalysts.^293,297^ In order to determine the true sample composition, a careful interpretation of XRD and XPS data or additional analytic techniques are necessary.

#### Cationic anionic codoped systems

N and La codoping is expected to reduce the formation of oxygen vacancies^280,312^ and impurity states in the band gap^313^ due to charge balance, furthermore it enhances the absorption ability and the photocatalytic activity of STO in visible light range.^280,312^ N,La:STO possesses one additional absorption band between 400 and 500 nm and features broad light absorption above 500 nm. Because of the codoping the oxidation of 2-propanol to CO_2_ is photocatalyzed above 410 nm and the catalytic activity in the UV range is not decreased compared to pristine STO.^312^ Other examples for mixed cationic and anionic codoping are C,Nb:STO^267^ and Cr,B:STO.^272^ First principle studies suggest that strong Coulomb interaction between donors and acceptors results in an enhanced stability of codoped systems.^313^

### 7.2 Heterojunctions

**Fig. 23:**
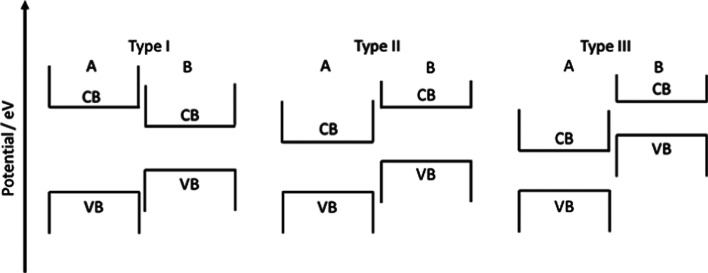
Schematic of the band position of type I, type II, and type III hetero junctions before contact is established.

Three types of heterojunctions can be distinguished, when two semiconductors (A and B) are brought into contact, compare Fig. 23. Depending on the respective band energies and doping levels, band alignment may lead to several different energetic situations with ohmic junctions or either charge carrier depletion or accumulation zones. Establishing a driving force for charge carrier separation is decisive for a junctions suitability for photocatalytic applications. Alternatives are metal/semiconductor heterojunctions, surface heterojunctions, and Z-scheme heterojunctions that mimic the natural photosynthesis based on two photon excitation processes. In the following, a few out of the various STO based heterojunction photocatalysts are explained.

**Fig. 24:**
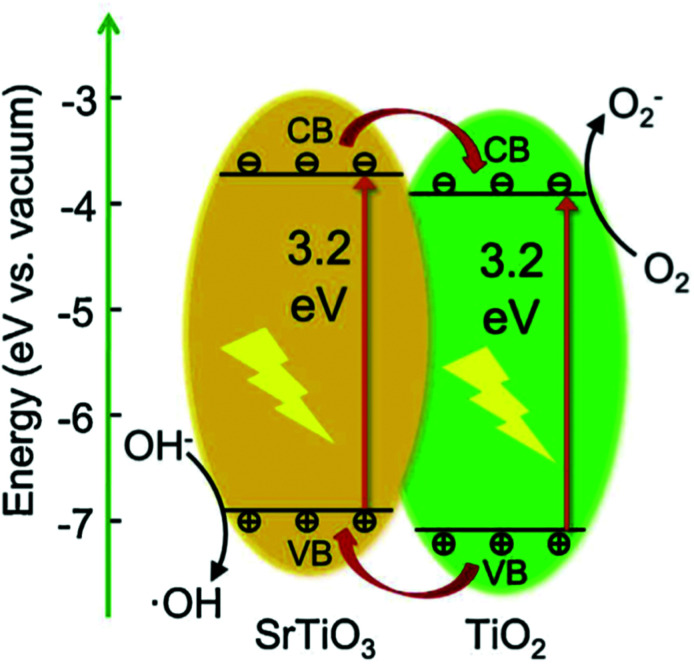
STO and TiO_2_ based type II heterojunction to illustrate the band positions and mechanisms responsible for the photocatalytic activity. Reprinted from ref. 242, with permission from Elsevier.

A widely used combination to form a type II heterojunction is STO/TiO_2_ with STO having higher conduction band and valence band energies.^220,232,242,244,314–321^ Holes are transferred from the TiO_2_ valence band to the valence band of STO. On the other hand, electrons move from the STO conduction band to the conduction band of TiO_2_ (see Fig. 24).^220^ A pure STO/TiO_2_ system only absorbs light in the UV range, since TiO_2_ and STO exhibit very similar band gaps of approximately 3.2 eV at room temperature. In order to facilitate/enable photocatalytic activity in the visible light range, STO and TiO_2_ can both be doped to modify the electronic structure, or their surface can be loaded with metal nanoparticles such as Pt, Rh, or Ru.^320^ An advantage of loading semiconductors with metals may be the formation of a Schottky barrier, further suppressing recombination *via* charge separation. However, if too many particles are present on the surface, they can also become recombination centers themselves,^320^ resulting in an optimal loading, *e.g.* in the case of Rh on STO, 0.05 wt%.^320^

A different way to enable visible light photocatalytic activity is to engineer a ternary system consisting of TiO_2_/STO/g-C_3_N_4_ (A) or STO/TiO_2_/g-C_3_N_4_ (B) nano compounds.^322,323^ In A, cascade charge transfer under UV illumination takes place, since the g-C_3_N_4_ valence and conduction bands are above the STO and the TiO_2_ valence/conduction bands. Excited electrons are transported to the TiO_2_ conduction band, whereas holes are transferred to the g-C_3_N_4_ conduction band.^323^ In B, electrons are still only moved to the TiO_2_ conduction band, but holes from the TiO_2_ valence band can now be transported either into the valence band of g-C_3_N_4_ or STO. Visible light can only excite electrons in g-C_3_N_4_, which exhibits a band gap of approx. 2.7 eV.^324^ Moreover, it is a cheap,^325^ polymeric and metal-free material^324^ and is chemically stable. The described ternary TiO_2_/STO/g-C_3_N_4_ has similar applications as TiO_2_/STO heterojunctions and can be used as photocatalyst in organic pollutant degradation,^242,314,320^ for hydrogen production^314,323^ or N_2_ fixation.^323^ The system can also be simplified to STO/g-C_3_N_4_.^247,273,283,325^ In such a two-component system it is easier to modify the electronic structure of STO, since in a composition with only one heterojunction, the position of the band gaps can be chosen more freely. To enable visible light absorption in both g-C_3_N_4_ and STO, doping of STO with Cr is common.^247^

Another example for a type II heterojunction for water splitting is NiO/STO.^218,224,326–328^ Under irradiation with a 450 W high pressure mercury lamp its water splitting ability is tremendously enhanced compared to pure STO.^232^ Moreover, it facilitates the evolution of H_2_ and O_2_ from pure water without the need of additional cocatalysts like Pt.^218^ A more recent study suggests that NiO_*x*_/STO compounds are in reality Ni/STO/NiO systems.^218^ On these, Ni^0^ acts as electron trap and as reduction site and lowers the proton reduction overpotential. NiO serves as water oxidation site, and limits the performance of this system.^218^

A heterojunction for methyl orange photodegradation can be achieved with the combination of La doped WO_3_ and STO. La:WO_3_ is non-toxic and exhibits a band gap of 2.8 eV, hence shows photocatalytic activity under visible light. STO serves as co-catalyst and increases the photocatalytic activity of La doped WO_3_. The degradation itself is caused by ˙OH and ˙O_2_^−^ radicals, holes, and hydrogen peroxide.^329^ In the last decades, many other STO/metal oxide type II heterojunctions such as STO/ZnO^330^ and STO/Ag_3_PO_4_^300^ were described in literature.

Metal sulfides such as CdS^221,243,331^ and MoS_2_^332^ can also form appropriate heterojunctions with STO. With the help of chemical bath deposition, CdS nanodots can be applied on STO nanocubes at room temperature. CdS/STO is able to facilitate hydrogen evolution under simulated sunlight irradiation. It exhibits an apparent quantum yield of 0.03% at 375 nm, which means an improvement by a factor of 12.2 compared to pure STO.^331^ The ideal loading of CdS on STO is reported to be approx. 23.6 wt%. CdS has a band gap of 2.38 eV and its conduction band is slightly lower than the STO conduction band. Consequently, electrons are transferred to CdS and the recombination of photogenerated charge carriers is suppressed.^331^

The MoS_2_/STO heterojunction can be used for organic dye degradation, since electrons from the STO conduction band can move to the lower conduction band of MoS_2_. Loadings of 0.05 wt% exhibited the best performance, higher amounts of MoS_2_ on the STO surface block the active sites.^332^ The absorption of STO does not change significantly due to MoS_2_ loading, hence dye degradation is only enhanced under UV illumination.^332^

An additional way to increase H_2_ production of STO based catalysts under UV light is the coating with a conducting hydrophilic organic compound. A heterojunction is formed and the absorption of H_2_O on the surface is improved. Consequently, charge recombination in the bulk and at the surface is reduced, furthermore the charge transfer from catalyst to the water molecule is alleviated.^333^

#### p–n junctions

**Fig. 25:**
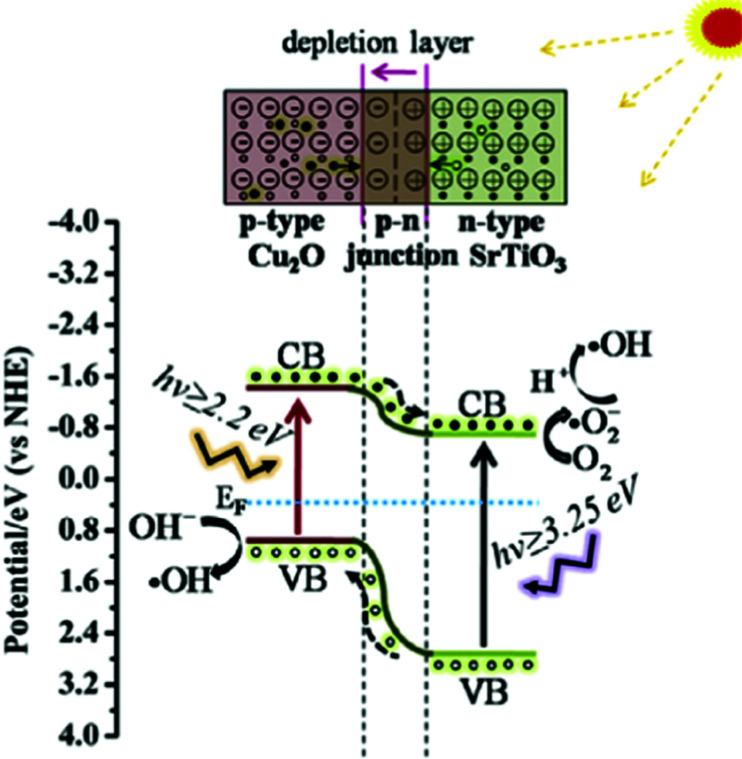
Schematic illustration of the n-STO/p-Cu_2_O p–n junction, with a built-in internal electric field, helping to separate charge carriers, thus suppressing recombination. Reprinted from ref. 334, with permission from Elsevier.

p–n junctions are a special form of type II heterojunctions as the additional internal field formed by the contact helps to further improve the photogenerated charge carrier separation. In the following, some examples of such systems are given and explained. CuO and Cu_2_O are p-type semiconductors and therefore form a p–n junction with n-type STO (compare Fig. 25). Thereby the Cu_*x*_O bands are shifted upwards. This leads to a synergistic effect and a photocatalytic activity in the UV and visible light range. Both the valence and the conduction bands of the copper oxides are above the corresponding bands of STO, consequently electrons are transferred to STO and holes to the copper oxides.^237,334–336^ Such composites can be used for CO_2_ conversion, H_2_ generation,^237,335^ or degradation of dyes.^334,335^

Bismuth oxyiodide, BiOI, is a p-type semiconductor with a band gap of 1.94 eV.^245^ It is catalytically active under visible light and stable but exhibits a fast recombination rate.^245^ In contact with STO, the Fermi levels align and the bands of the BiOI nanoparticles on STO are shifted upwards, causing an electric field, enhancing charge carrier separation and suppressing recombination.^245^ STO/BiOI is able to catalyze the degradation of organic pollutants such as bisphenol A or methyl orange. The involved reactive species in this process are mainly h˙ and ˙O_2_^−^.^245^

#### Metal loading on the STO surface

Metal surface loadings of Cu,^235,296^ Ir,^264^ Pt,^216,308,320,337^ Rh,^320^ Ru,^320^ Au,^221,338^ and Ag^227,240,250,339,340^ on STO and their influence on the photocatalytic activity have been thoroughly investigated over the last decades. The metals typically act as electron sink and as active reaction sites, thereby enhancing the separation of photogenerated electrons and holes. Too high loading amounts lead to overlapping agglomerations and possibly to electron accumulations, which induce an electric field which attracts electron holes.^240,320^

Pt is the most commonly used loading material for photocatalytic water splitting and organic pollutant degradation. Pt nanoclusters form a Schottky barrier with STO, facilitate the reduction process and absorb visible light due to localized energy levels in the band gap of STO. However, studies suggest that under optimal conditions, Cu can be a cheaper replacement for Pt. STO loaded with 0.5 wt% Cu shows a similar performance as STO loaded with 0.5 wt% Pt in the H_2_ evolution from aqueous methanol solution under UV irradiation.^235^ Similar to metal loadings, graphene can also act as an electron sink and enhance the charge separation.^341^

Additionally, Au,^342–345^ Ag^339,340,345,346^ and Pd^347^ nanoparticles are known to exhibit surface plasmon resonance (SPR), which can be capitalized in photocatalytic processes. Free charge carries collectively oscillate and when light with the oscillation frequency shines on the nanoparticles, strong absorption can be observed. This resonance frequency can be tuned by variation of material, surrounding, shape, and size of the metal clusters. The smaller the nanoparticles, the lower the resonance frequency becomes. Ag clusters can thereby be tuned to absorb light in the visible range and gold clusters even in the near infrared. Light below the resonance frequency is reflected and light with a wavelength too short for absorption is transmitted.^345,348^

Three mechanisms are discussed to improve the photocatalytic activity of metal semiconductor heterojunctions, based on surface plasmon resonance.^349^ Firstly, electrons are easily excited in Au or Ag by plasmon resonance and subsequently transferred by tunnelling to the conduction band of the nearby semiconductor. For this electron injection, the metal and the semiconductor have to be in direct contact. Secondly, huge and strongly localized electric fields are generated by the light absorption. In areas very close to these hot spots, the photogeneration of electron hole pairs is strongly accelerated in the semiconductor. Moreover, the field intensity is spatially inhomogeneous, the closer to the metal surface, the stronger the electric field becomes. This has two main impacts, (i) the photogenerated electrons holes are separated (suppressed recombination) and (ii) most of the charge carries are formed close to the semiconductors surface (short migration length). Thirdly, photons are scattered by the metal clusters. This causes an increased electron hole pair formation since the mean photon path is increased.^349^ Since the resonance frequencies are in the range of visible light and undoped STO exhibits a band gap of approx. 3.2 eV at room temperature, Au or Ag loading alone would not lead to an optimal enhancement, since the incoming photons need to induce the surface plasmon resonance effects and excite electrons in the semiconductor. Consequently, to utilize the surface plasmon resonance effect, the metal nanostructures have to be carefully designed and the metal loading needs to be combined with doping of STO.^345^

#### Z-Scheme systems

In contrast to other approaches, Z-Scheme systems are based on a two-photon process and two photocatalysts are combined. One is specially designed for the oxygen evolution reaction and the other for the hydrogen evolution reaction in an overall water splitting system. Between the two, usually a redox system (redox mediator) is responsible for the uptake of photogenerated electrons from the conduction bands of the oxygen evolution photocatalyst (OEP) and the photogenerated holes from the valence band of the hydrogen evolution photocatalyst (HEP), thus enabling an electric current. The valence band of the OEP is more positive than the H_2_O/O_2_ potential whereas the conduction band of the HEP is so negative that it facilitates the proton reduction. Optionally each catalyst surface may be loaded with co-catalysts such as Ru, Mo, or Pt.^223,261^

**Fig. 26:**
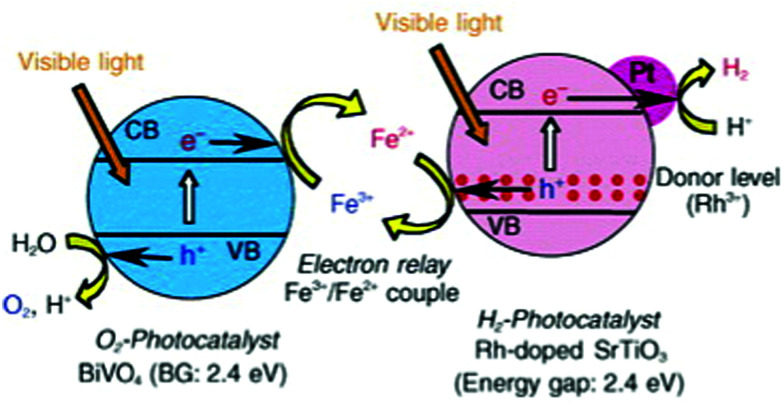
Band position and schematic of the mechanism behind a Z-scheme photocatalyst based on Pt loaded Rh doped STO, BiVO_4_, and the Fe^2+/3+^ couple as mediator. The energy gap between Rh^3+^ donor levels and the CB of STO amounts to 2.4 eV, hence visible light is able to excite e^−^ from the valence band to the conduction band. Rh:STO acts as HEP and BiVO_4_ as OEP. Reprinted from ref. 216 with permission from the Chemical Society of Japan.

Such Z-scheme photocatalyst systems can be rather complicated (compare Fig. 26), and STO based systems still exhibit moderate solar to hydrogen energy conversion efficiencies, the best in the range of 1.2% at 420 nm, 331 K, and under 10 kPa.^261^ However, they have some undeniable advantages such as: the catalysts can be individually optimized for the oxidation and reduction reactions, the oxygen and hydrogen evolution can be spatially separated, and visible light is sufficient to excite electrons from the conduction bands to the valence bands.^261,303^

In the last years, the most promising HEPs have been La,Cr:STO,^274^ La,Rh:STO,^223^ Cr,Ta:STO^309^ or Rh:STO.^304,305,337,350^ All of these can be loaded with cocatalysts, for example Ru^222,303–305,350^ or Pt.^302,309,337^ The loading takes care of the electron transfer to reduce H^+^ and can enhance the charge separation. Rh doping leads to the formation of Rh^3+^ and Rh^4+^ and enables STO to absorb visible light, due to the introducing Rh^3+^ electron donor levels.^222,302,303^ As redox mediator between the HEPs and OEPs, various systems were developed and have their specific advantages and disadvantages. In general, they need to meet certain criteria to be suitable: their redox potential must be between the reduction and oxidation potential of water, they have to be transparent and the change of their oxidation state has to reversible under the predominant conditions. Used mediator systems are IO_3_^−^/I^−^,^309,351^ Fe^2+^/Fe^3+^,^302,304,337,351^ [Co(bpy)_3_]^3+^/[Co(bpy)_3_]^2+^,^222,303^ [Co(phen)_3_]^3+^/[Co(phen)_3_]^2+^ ^303^ and VO_2_^+^/VO^2+^.^352^ All of these operate in aqueous solutions with pH ranging from 2.1 to 11. Cobalt complexes exhibit a high turnover rate, bind well to the catalysts surfaces, are very selective to the forward reaction and have their optimum at pH 7. In acidic reaction solutions, the oxygen evolution is hindered and instead Co^2+^ is oxidized. IO_3_^−^/I^−^ and Fe^2+^/Fe^3+^ systems are widely-used and Fe^2+^ can also act as a catalyst for the H_2_ production. On the other hand, Fe^3+^/Fe^2+^ systems can only be used up to approx. 550 nm. In a Rh:STO based and Fe^2+^/Fe^3+^ mediated system, Ru loading leads to a higher photocatalytic activity than Pt loading, since the suppression of the backward reaction is improved by Ru.^261^

Additionally to the listed redox mediators, also solids such as Au,^353^ Ag,^250^ Ir,^281^ indium tin oxide^223^ and reduced graphene oxide^350^ can act as mediators, however the synthesis of such systems is challenging. Some studies went even further and abandoned the mediator completely. In these, for example reversible Rh^3+/4+^ species on the surface of Rh doped STO act as charge carrier/transporter.^305^

As OEPs BiVO_4_,^302,304,305^ BiVO_4_ loaded with RuO_2_, Mo^353^ or CdS,^354^ WO_3_,^274^ WO_3_ with PtO_*x*_^355^ or Pt^355^ loading, AgNbO_3_,^261^ Cr,Sb:TiO_2_,^261^ Ta_3_N_5_ loaded with CoO_*x*_,^281^ Bi_2_MoO_6_,^216,261^ Bi_6_NbWO_14_Cl,^261,356^ Ag_3_PO_4_^250^ and others^248^ can be found in literature. WO_3_ and BiVO_4_ do not need any co-catalyst in combination with an Fe^3+^/Fe^2+^ mediator for the oxygen evolution. Moreover, these two are the most common OEPs in STO based Z-scheme photocatalysts.^261^

### 7.3 Further techniques

#### Surface anisotropy

As described above, charge separation is one of the main challenges during photocatalytic processes and many strategies were developed to suppress charge recombination. In most approaches, metal loadings are used as electron sinks and/or enhance the charge separation by forming a Schottky barrier. However, also the surface anisotropy between the {001} and {110} facets of STO can be capitalized to increase the photocatalytic activity. The {001} surface is charge neutral, due to its SrO or TiO_2_ termination. The {110} facets are formed either by positively charged STO or negatively charged oxygen layers. Furthermore, under illumination with wavelengths near the absorption edge, the electronic band structure of STO facilitates the formation of electrons and holes with a momentum in the [001] direction. This may be the reason that the {001} facet exhibits a higher photocatalytic activity than the {111} or {110} facets.^357^ In general, the existence of catalytic anisotropy as well as oxidizing and reducing facets is known not only for STO,^358,359^ but also for BiVO_4_,^360,361^ TiO_2_,^362^ and Cu_2_O.^363^

**Fig. 27:**
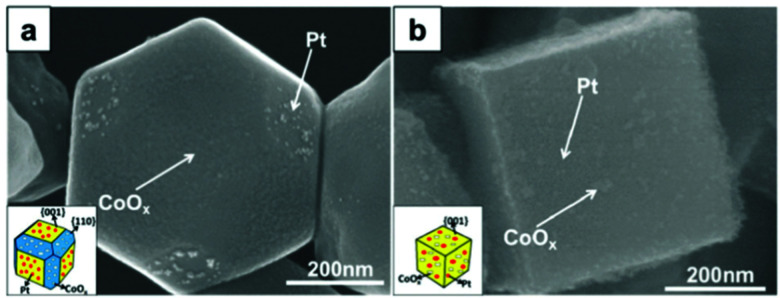
(a) 18-Facet STO crystal with spatially separated Pt and CoO_*x*_ cocatalysts. (b) Cubic STO with only {001} facets present and cocatalysts distributed randomly. Reprinted from ref. 358, with permission from the Royal Society of Chemistry.

18-Facet STO microcrystals were produced and a cocatalyst was selectively photodeposited either on the {100} or the {110} facets. On the {100} surfaces, Pt nanoparticles facilitate the hydrogen evolution and on the {110} surface, Co_3_O_4_ facilitates the oxygen evolution in overall water splitting. Such systems with two spatially separated cocatalysts show a five-time better quantum efficiency than STO with randomly distributed cocatalysts on its surfaces (in the case of cubic STO, with only six {100} facets present).^358^ In Fig. 27, SEM images of STO nanocrystals with either randomly distributed or separated cocatalysts are shown. Another study showed that charge and cocatalyst separation is even possible on one surface of an STO cube. The authors suggest that the upwards surface band bending depends on illumination and is stronger at the edges and corners under no or under weak irradiation. This leads to a hole movement towards the edges and corners and to the ability to deposit Pt selectively in the center and Co_3_O_4_ on the edges of STO cubes.^364^

#### Surface manipulation

Carboxyl groups from oleic acids can bond to Ti^4+^ on STO and by forming a dipole layer, visible light absorption for photocatalytic NO degradation becomes possible.^365^ Surface-alkalization of STO in a NaOH/methanol/water solution with pH > 13 shifts the surface energy bands upwards. Thus, reduction power is enhanced and the decreased oxidation potential is balanced by the fact that the absorbed hydroxide ions promote the methanol oxidation.^366^ Sc doped STO with Rh_2_O_3_ as cocatalyst suffers from a fast back reaction of H_2_ and O_2_ acting as a chemical short circuit. Here, semipermeable protective layers consisting of Cr_2_O_3_ or Ta_2_O_3_ covering the cocatalysts and the STO surface block oxygen and organic molecules (*e.g.* ethanol) from active sides, whereas H_2_O and H_2_O_2_ can reach the surface of the catalyst. Consequently the total photocatalytic activity of overall water splitting is increased.^275^

Oxygen vacancies near the STO surface can act as electron traps and adsorption sites for N_2_, hence enhancing photocatalytic activity for N_2_ fixation.^255^ A different thermal expansion coefficient of STO and Au when combining the materials, can be used to introduce tensile strain in STO and thus promote the formation of oxygen vacancies and Ti^3+^, altering the electronic band structure and thus enhancing the photocatalytic activity of STO.^367^ Further, also liquid nitrogen treatment may introduce compressive strain, causing a narrowed band gap and consequently a shifted light absorption edge to lower energies.^255^ Solid state reaction of STO with NaBH_4_ leads to a disordered shell while retaining a crystalline bulk. Again, oxygen vacancies are introduced and the oxygen vacancy concentration for optimal efficiency was found to be in the range of 3.3 at%.^368^ Treating STO with NaBH_4_ was further beneficial by increasing light absorption in the visible light range.^368^

## 8 Magnetic properties

STO is commonly known as a diamagnetic material, however, it has recently drawn renewed attention to its magnetic properties due to the discovery of ferromagnetism in certain samples.^107,369–377^ While the origin of this ferromagnetism is still not fully resolved, it is secured, that defects play a major role in the source of these magnetic properties.^369,371,376^ Lee *et al.* discovered, that Mn and Co doped STO exhibits room temperature ferromagnetism,^378^ however, they do not elucidate the mechanics behind this effect. Sikam *et al.* consequently performed DFT calculations for Co doped STO and found that ferromagnetism is, on the one hand, induced directly by spin inequalities introduced by the Co doping itself and, on the other hand, potentially affected by the presence of oxygen vacancies in the lattice.^379^ Middey *et al.* correlated the magnetic properties of Mn doped STO at low temperatures directly to the presence of oxygen vacancies, as samples without oxygen vacancies are fully paramagnetic, while after introduction of oxygen vacancies a ferromagnetic hysteresis loop is observed.^373^ Trabelsi *et al.* found the same effect for undoped STO,^370^ while Xu *et al.* observed ferromagnetism in oxygen deficient STO thin films,^107^ thus backing the assumption that oxygen vacancies alone are a driving factor for ferromagnetic properties of STO.

This assumption was thereafter confirmed by several independent DFT calculations, observing ferromagnetic properties for different degrees of oxygen deficiency and identifying Ti states in the surrounding of oxygen vacancies as the underlying cause.^369,371,376,377,380^ However, there is still no broad agreement about the exact nature of the oxygen vacancies causing ferromagnetic properties in STO with regard to clustering, their location at the surface or inside the bulk and to their charge state.^372,375,376,380–382^ Additionally, research also focusses on the role of titanium vacancies and on the effect of lattice strain on the magnetic properties of STO.^369,377,383^

**Fig. 28:**
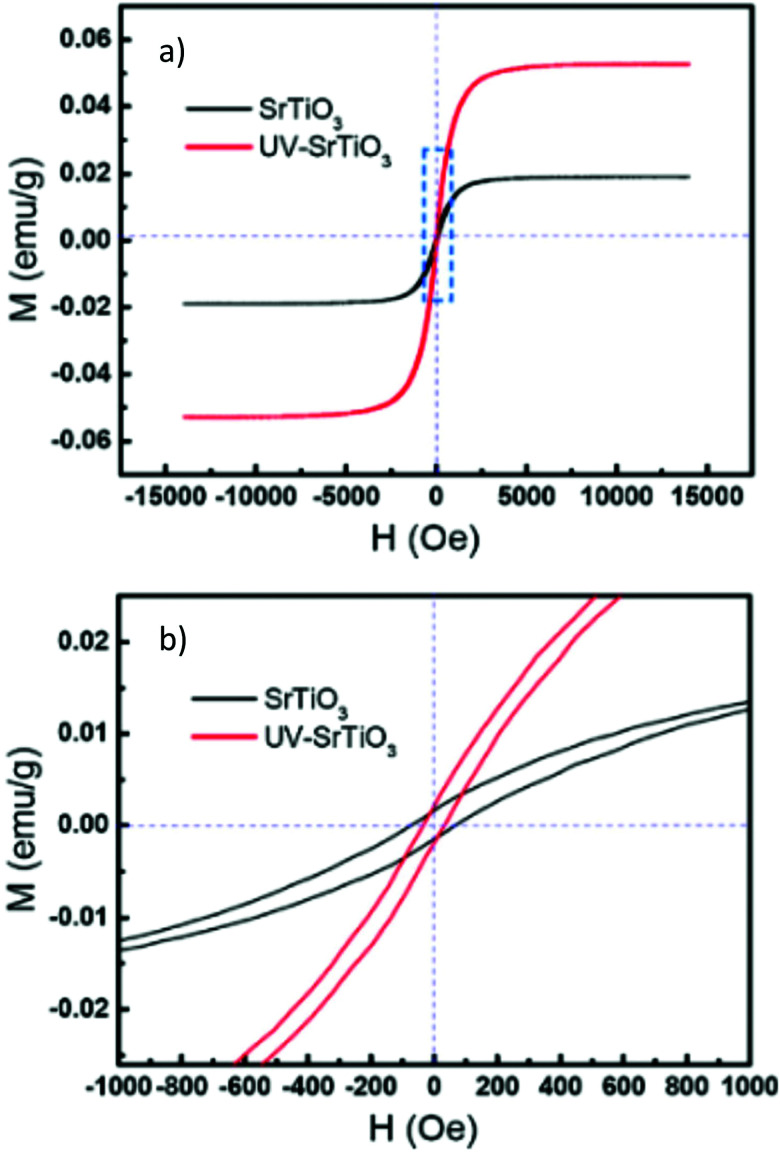
(a) Hysteresis loops of the STO sample and the sample after UV irradiation measured at room temperature. (b) The enlarged part in the center of the loops in (a). Reprinted from ref. 384, with permission from Elsevier.

This strong correlation of the magnetic properties of STO with oxygen vacancies suggests that UV illumination could have a significant effect on magnetism in STO. Indeed, Zhang *et al.* observed, that the ferromagnetic response of STO nanocubes is significantly enhanced after UV illumination at room temperature^384^ (see Fig. 28). This enhanced ferromagnetism is correlated with an increased density of oxygen vacancies, introduced during the illumination process. Similar behaviour was also found by Qin *et al.* in the closely related BaTiO_3_, where the increase of the saturation magnetization is even larger at a factor of 10.^385^

For the sake of completeness we do not want to exclude research on the photoresponse of magnetic properties of STO-based heterostructures. For instance, Katsu *et al.* report reduced magnetization of STO/(La,Sr)MnO_3_ heterostructures,^386,387^ while Jin *et al.* report the suppression of the Kondo effect at STO/LaAlO_3_ interfaces under UV illumination.^388^ However, detailed reviews of the photoresponse of STO-based heterostructures are available.^85,86^

## 9 Conclusions and perspectives

With STO being a model system for the investigation of the fundamentals of wide band gap semiconductors, great efforts have been directed towards the understanding of the electronic structures of STO and the effect of defects on its properties. Especially the interaction of STO with light is a powerful access point to its defect chemistry and many phenomena have been described and investigated deeply. Moreover, photoinduced processes are relevant in many up-and-coming fields of applications and show great potential for future technologies. However, it is precisely the great variety of different defects which adds a layer of complexity to all observations in this field, and specific defects are often hard to identify as they occur in low concentrations or in the form of associates and trapping effects can complicate the landscape of electronic charge carriers.

Recent efforts in the field of high temperature measurements further show that the ionic and electronic properties are inseparably linked and many photoinduced effects observed at low temperatures also appear at high temperatures, however with fundamentally different underlying mechanisms. While light interacts with the local electronic structure of STO at low temperatures, the increased oxygen vacancy mobility at higher temperatures together with the capability of STO to accommodate a certain non-stoichiometry lead to the observation of similar phenomena, like photoconductivity, photochromism or photovoltages, but all based on very different processes. Despite the different mechanisms, also at high temperatures, defects, impurities and particularly their concomitant electronic trapping energies play an essential part in the specific phenomena observed under illumination.

These recent results increasingly emphasize a major point of action for future research, as it is necessary to characterize these defects and their impact on the electronic structure in great detail to understand the processes induced by irradiation. Here, limits are often set by analytical techniques as relevant defect concentrations are often in the ppm range and below. However, elaborate analytical methods combined with defect chemical considerations could reveal the fundamental interactions of light with STO and its defect chemistry and pave the way for its broad implementation in energy and sensoring applications.

Currently, light-based applications are often limited by the wide band gap of STO and the restriction to ultraviolet light, as well as by a limited understanding of the physics of STO surfaces and STO-based interfaces. For example, the photocatalytic activity of SrTiO_3_ has been improved drastically by doping, the formation of heterojunctions, and surface engineering. However, an improved suppression of recombination centers and an optimal band alignment in heterojunction materials could potentially increase the photocatalytic capability of SrTiO_3_ even further, giving a clear direction for research towards stable, scalable and efficient photocatalytic systems. With regard to photovoltage-related applications, a variety of different top layers on Nb:STO exist, ranging from different manganites to yttrium barium copper oxide to various other oxides. Here, for manganite top layers, a lot of research has already been carried out, but a more systematic approach is needed to advance the field of oxide photovoltaics, while for high temperature photovoltaics, the complexity of mixed ionic and electronic interactions at interfaces engages multidisciplinary research efforts and challenges the exploration of uncharted scientific territory. Concerning photoconductivity and photoluminescence, the biggest issue in today's research is the aforementioned identification and quantification of specific defects, especially since the defect chemistry of STO can vary significantly for different doping levels and sample preparation methods. Here, a more systematic approach is needed, to correlate an *a priori* known defect situation with certain phenomena like persistent photoconductivity or specific luminescence bands.

In conclusion, the understanding of photoinduced effects in strontium titanate has been advanced considerably over the last decades and especially during the last few years, uncovering new types of interaction and pinpointing different effects to particular defects. A broad overview of the existing research shows that in many cases, the sheer multitude of possible defects responsible for the observed effects impedes a systematic investigation. However, these limits have been continuously tested and many have been overcome on the way to a comprehensive understanding of the defect chemistry of STO and to its application in future technologies.

## Conflicts of interest

There are no conflicts of interest to declare.

## Supplementary Material
